# Anatomically Designed Triboelectric Wristbands with Adaptive Accelerated Learning for Human–Machine Interfaces

**DOI:** 10.1002/advs.202205960

**Published:** 2023-01-22

**Authors:** Han Fang, Lei Wang, Zhongzheng Fu, Liang Xu, Wei Guo, Jian Huang, Zhong Lin Wang, Hao Wu

**Affiliations:** ^1^ Flexible Electronics Research Center State Key Laboratory of Digital Manufacturing Equipment and Technology School of Mechanical Science and Engineering Huazhong University of Science and Technology Wuhan 430074 China; ^2^ Ministry of Education Key Laboratory of Image Processing and Intelligent Control School of Artificial Intelligence and Automation Huazhong University of Science and Technology Wuhan 430074 China; ^3^ Beijing Institute of Nanoenergy and Nanosystems Chinese Academy of Sciences Beijing 101400 China; ^4^ School of Materials Science and Engineering Georgia Institute of Technology Atlanta GA 30332‐0245 USA

**Keywords:** flexible electronics, gesture recognition, human–machine interfaces, machine learning

## Abstract

Recent advances in flexible wearable devices have boosted the remarkable development of devices for human–machine interfaces, which are of great value to emerging cybernetics, robotics, and Metaverse systems. However, the effectiveness of existing approaches is limited by the quality of sensor data and classification models with high computational costs. Here, a novel gesture recognition system with triboelectric smart wristbands and an adaptive accelerated learning (AAL) model is proposed. The sensor array is well deployed according to the wrist anatomy and retrieves hand motions from a distance, exhibiting highly sensitive and high‐quality sensing capabilities beyond existing methods. Importantly, the anatomical design leads to the close correspondence between the actions of dominant muscle/tendon groups and gestures, and the resulting distinctive features in sensor signals are very valuable for differentiating gestures with data from 7 sensors. The AAL model realizes a 97.56% identification accuracy in training 21 classes with only one‐third operands of the original neural network. The applications of the system are further exploited in real‐time somatosensory teleoperations with a low latency of <1 s, revealing a new possibility for endowing cyber‐human interactions with disruptive innovation and immersive experience.

## Graphical abstract

Anatomically designed triboelectric wristbands that can interpret human intention are described. The anatomical design contributes to enhanced classification accuracy due to the close correspondence between the actions of dominant muscle groups and gestures. The adaptive pruning strategy can aggressively reduce the computational operands with trivial loss of accuracy. Consequently, 97.56% accuracy is realized by only 7 sensors for 21 gestures.

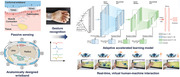

## 1 Introduction

Smart wearable technologies are rapidly revolutionizing users’ experiences in various technological fields, such as human‐robot interfaces (human–machine interfaces (HMIs)), robotic tactile sensing, personalized healthcare, virtual/augmented reality, etc.^[^ ^1^, ^2^, ^3^, ^4^ ^]^ Wearable flexible electronics are increasingly prevalent in HMI applications due to their lightness and comfort.^[^ ^5^, ^6^ ^]^ Moreover, wearable human gesture recognition (HGR) systems for remote control of robotic systems are of particular interest due to the versatile commands human hands can order. With the remarkable progress in burgeoning technologies (such as 5G/6G communication,^[^ ^7^ ^]^ extended reality (XR),^[^ ^8^ ^]^ and digital twins^[^ ^9^ ^]^) and the influence of the current coronavirus disease (COVID‐19) pandemic, our life is migrating to the noncontact “Metaverse” society, a virtual online world that maps and interacts with the physical reality.^[^ ^10^ ^]^ The noncontact culture further accelerates the exigent demands for remote forms of immersive user interactions. By introducing new soft functional materials and wearable electronics to the Metaverse research field, diverse transduction methods, such as the piezoelectric effect,^[^ ^11^ ^]^ piezoresistive effect,^[^ ^12^ ^]^ ionic conduction,^[^ ^13^ ^]^ and capacitive effect,^[^ ^14^ ^]^ are under development for decoding human intentions. However, the most limitations of these mechanisms lie in the demand of external power suppliers, inhibiting their mass production and widespread use.^[^ ^15^ ^]^ Recently, triboelectric nanogenerators (TENGs) have emerged as low‐cost and self‐sustainable wearable HMIs by scavenging ubiquitous energy from human movements.^[^ ^16^, ^17^, ^18^, ^19^ ^]^ The advantages of wide material choice, lightweight, simple manufacturing, high output, and expeditious dynamic response render TENGs suitable for capturing mechanical stimuli of low frequencies and minute amplitudes.^[^ ^20^, ^21^, ^22^ ^]^ To accurately interpret human intention, it is critical to detect sophisticated motions of dexterous hands with sensitivity and effectiveness. Finger‐bending/touching triboelectric sensors integrated with gloves or exoskeletons have been developed in past years.^[^ ^23^, ^24^, ^25^, ^26^ ^]^ Nonetheless, the main restrictions of those systems are the complicated and rigid frameworks, which could hinder precise hand motions and cause poor wearing comfort (e.g., due to sweating). In addition, these prototypes need complex sensor networks covering the entire curvilinear area of the hand or forearm to pinpoint the motions of every joint or muscle, embodying inefficiencies in both sensing and gesture classification processes.

An alternative approach for capturing gestures effectively and conveniently is indirectly tracking forearm muscle activities relevant to hand motions.^[^ ^27^ ^]^ Generally, surface electromyography (sEMG),^[^ ^28^, ^29^ ^]^ inertial navigation,^[^ ^30^ ^]^ and visual image/video processing^[^ ^31^ ^]^ are dominant strategies. Among them, sEMG‐based wearable prototypes, such as wristbands and armbands, have been extensively investigated for reconstructing the action potential along myocytes into control commands in HMIs.^[^ ^30^, ^32^, ^33^ ^]^ However, the thorny challenges for sEMG‐based systems with weak signal strength (in microvolts), poor quality sensor data, susceptibility to surrounding interference, crosstalk with other biopotentials, and large power consumption remain unresolved.^[^ ^34^ ^]^ In addition, existing sEMG‐based systems are usually dependent on precisely positioned, bulky electrodes that are susceptive to electromagnetic noise and varying physiological conditions (such as sweating and fatigue).^[^ ^35^, ^36^ ^]^ To avoid device doffing and replicate the faithful information, the sEMG‐based band needs to tightly hold the forearm in a vice‐like grip, which will induce strong oppression on the forearm and skin discomfort during long‐time use.^[^ ^30^, ^33^ ^]^ Moreover, it is difficult to perform sEMG sensing at the wrist, because there are relatively fewer myocytes and denser distal tendons (near the wrist) compared with the proximal upper forearm (near the elbow).^[^ ^32^, ^37^ ^]^ Complementary to traditional approaches, highly‐sensitive and straightforward triboelectric wristbands could be a promising assistive platform, which is immune from the problems of the sEMG‐based prototypes encountered above. By leveraging the rapidly developed machine‐learning models, intelligent wristbands can distinguish sophisticated and similar hand motions with high classification accuracy and unlock diversified somatosensory operations.

Different machine learning methods have been employed in motion classification from recorded multi‐channel signals, such as support vector machine^[^ ^24^ ^]^ and K‐nearest‐neighbor^[^ ^38^ ^]^ classifiers, as well as deep convolutional neural networks (CNNs).^[^ ^25^, ^26^ ^]^ In particular, a CNN can implicitly and automatically extract deep features from time‐series signals,^[^ ^31^ ^]^ heralding a promising solution for realizing better classification functions. However, processing sophisticated and similar data typically involves huge sliding convolutional operations depending on a wider and deeper CNN architecture.^[^ ^39^ ^]^ As a result, the soaring model parameters will affect the training efficiency, and their implementation on mobile terminals becomes infeasible due to limited resources.^[^ ^40^ ^]^ In addition, the increased latency generated by complicated models will cause poor user experience in real‐time human–machine interactions. Alternatively, lightweight network design for accelerating the computing procedures with reduced convolutional models has become one of the important CNN optimization methods.^[^ ^41^, ^42^ ^]^ Nevertheless, the redesigned models with smaller sizes may lead to suboptimal performance—that is, the CNN structure is selectively preserved or eliminated by evaluating its importance, and the classification results are reliant on sparse datasets.^[^ ^31^, ^39^ ^]^ Therefore, the ability to construct a highly‐efficient sampling procedure while maintaining the property of high generalization is desirable.

In this article, we explore the passive response of triboelectric sensors to mechanical deformation from wrist muscle/tendons and identify sophisticated hand motions with adaptive accelerated learning (AAL). Self‐powered, flexible, low‐cost, and noninvasive sensor array can conform to the skin of the human wrist with the assistance of an adjustable strap. The sensor array is deployed according to the wrist anatomy for detecting multiple finger and wrist motions from a distance. Particularly, the correspondence between the actions of dominant muscle/tendon groups and gestures results in distinctive features in sensor signals, which contributes to enhanced classification accuracy. The output electrical signals exhibit high sensitivity, fast response time, high quality, and excellent durability, revealing a promising method to clarify human intention that transcends the traditional sEMG. By leveraging an adaptive pruning strategy, the deep training model is aggressively compressed and updated in response to different inputs. As a result, the computation cost of the sub‐model is reduced to 37.78% of the original neural network, and test accuracy of 97.56% can still be reached in training 21 hand motions based on the data from only 7 sensors. The synchronized multi‐channel triboelectric signals can be precisely recognized as different HMI commands, which are wirelessly sent to multiple targets to project real‐time somatosensory teleoperations with a short delay of < 1 s. The findings in this work offer an aspirational approach for noncontact HMI with a flexible, portable, and highly‐efficient gesture interface, paving the way to the realization of next‐generation multifunctional technology in the near future.

## 2 Results and Discussion

### 2.1 Anatomical Design of the Gesture Recognition System and Wristband Configuration

**Figure 1:**
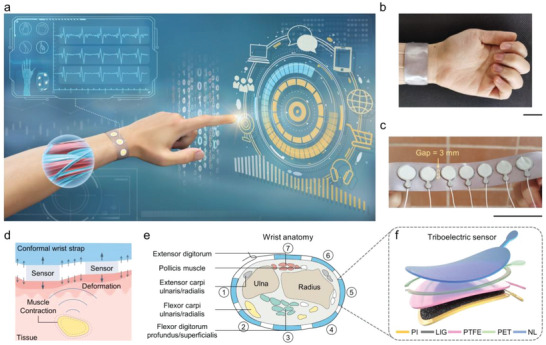
The schematics, structure, and anatomical design principle of the gesture recognition system enabled by a triboelectric wristband. a) Schematic illustration of the gesture recognition system. b,c) Optical images of a triboelectric smart wristband equipped with 7 sensors, which can conform to the human wrist. Scale bars, 5 cm. d) Passive triboelectric sensor for monitoring muscle/tendon contraction. e) Cross‐section anatomy of the human wrist, in which the anterior of the forearm is at the bottom. Channels are ordered from the surface nearest the ulna (channel 1), with an increasing number of channels wrapping around the anterior side of the forearm to the surface nearest the posterior extensor muscle (channel 7). The fastening strap is arranged between channels 1 and 7. f) Schematic structure of the triboelectric sensor.

As a proof‐of‐concept demonstration, we developed triboelectric smart wristbands with hand motion‐sensing capabilities (**Figure** **1**a). With dynamic hand motions, the triboelectric sensor array detects the topographical deformation of the wrist skin and generates a series of electric signals. Through leveraging a machine learning‐assisted classifier, the gesture recognition system can recognize individual motion as a specific command for multi‐class HMIs. With the aid of a slender wrist strap, circular‐shaped triboelectric sensors can be conformally laminated on the wrist for motion detection (Figure 1b,c). The wrist strap is made of silk materials with great flexibility, breathability, and comfort, assisting in the locating and fixation of sensors. To fully cover the muscle/tendon groups on the wrist, we deploy 7 sensors with an adjacent distance of 3 mm. The sensor size can be customized since the coverage area differs for individuals. Moreover, each sensor can be easily removed and replaced without damaging the overall structure, which ensures the high interchangeability of the sensing system. Compared with other standalone gesture recognition systems that are in the form of a glove (26), arm sleeve,^[^ ^43^ ^]^ patch,^[^ ^44^ ^]^ or exoskeleton,^[^ ^18^ ^]^ our wearable system is more portable and ergonomic, leaving less wearing burdens to the user and will not hinder any hand motion.

The triboelectric sensor operates as passive sensing that probes the mechanical skin deformations induced by muscle/tendon contraction in deep tissue (Figure 1d).^[^ ^45^ ^]^ Since the position of sensors is critical to identify specific muscle/tendon movements, the sensor distribution needs to be optimized based on the anatomical study of hand musculature. The human hand is articulated by intricate musculature to accomplish precise and versatile manipulations.^[^ ^27^ ^]^ As sketched in Figure 1e and Figure S1, Supporting Information, each muscle/tendon for controlling movements of digits and wrist exhibits a complex path, which is often interconnected at the wrist position. Therefore, there are increased challenges in predicting hand motions by sensing at the wrist than at the upper forearm.^[^ ^32^ ^]^ The flexion and extension of the digits (except the thumb) are mainly coordinated by the anterior flexor muscles (top red dots in Figure 1e, closer to the epidermis) and posterior extensor muscles (bottom green dots, tightly bound, and deeper), respectively. In contrast, the movements of the thumb are more independent, which are motivated by the scattered pollicis muscle groups (white dots). The flexor carpi ulnaris/radialis (yellow dots, bottom left/right) and extensor carpi ulnaris/radialis (grey dots, top left/right) control the motions of the entire hand at the wrist joint. Accordingly, the triboelectric sensors are attached above each muscle/tendon group for sensing skin deformations induced by relevant muscle/tendon movements. In particular, sensors 1, 3, 5, and 7 located in the orthogonal directions are of great importance in discriminating the wrist joint's four translational and rotational motions.

Figure 1f depicts the structure of the triboelectric sensor. The sensor employs the typical single‐electrode mode using laser‐induced graphene (LIG) as the electrode, which is scribed on a thin polyimide (PI) substrate. Polytetrafluoroethylene (PTFE) and natural latex (NL) films are distributed on both sides of a polyethylene terephthalate (PET) annular spacer, which breaks at the polar position to reserve a small space as an air‐breathing channel. The sensor is packaged with an insulated natural latex film that directly contacts the skin near the wrist, which can protect the electrification area from the contamination of sweat, dust, and skin flakes, as well as the interference of skin surface charges (Figure S2, Supporting Information). All of the materials used to construct the key components of the sensor are low‐cost and obtained via an inherently scalable and cost‐effective approach, facilitating the mass manufacturability and reproducibility of the electronics. The fabrication details are described in Section 4 and Figure S3b, Supporting Information. Owing to the use of soft and thin materials (for the optical image of each component see Figure S3a, Supporting Information), the sensing unit can conform to human skin and the deformation of muscle/tendons at the wrist.

### 2.2 Characterizations of the Triboelectric Sensor

**Figure 2:**
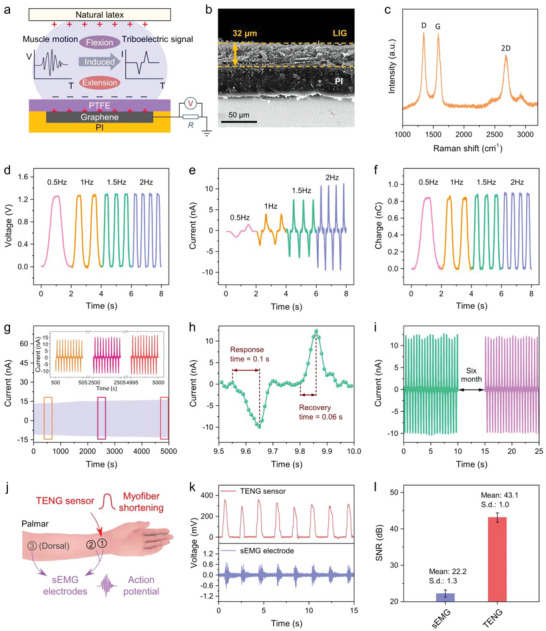
Characterizations of the triboelectric sensor. a) Schematic illustration of the triboelectric sensor for muscle/tendon motion detection and the measured mode of electrical signals. b) Cross‐sectional SEM image of the LIG film on PI sheet. c) Raman spectrum of the LIG film. a.u., arbitrary units. d–f) The *V*_oc_, *Q*_sc_, and *I*_sc_ output dependence on applied frequencies (from 0.5 to 2 Hz) of a force of 1 N. g) Mechanical durability test that lasted for ≈10 000 cycles. Inset: The *V*_oc_ output signals generated for the beginning (500–505 s), in the middle part (2500–2505 s), and in the final 5 s. h) Response and recovery time measured in g) (9.5–10.0 s). i) Long‐term stability of the triboelectric sensor after storage in the dry environment for six months. j) Schematic illustration showing the setup for monitoring the anterior flexor muscles. k) Corresponding output voltage signals obtained from the triboelectric sensor and sEMG electrodes, respectively. l) The SNR value of the triboelectric sensor (garnet) is much higher than that of sEMG electrodes (purple).

As shown in **Figure** **2**a, the fabricated triboelectric sensor can actively capture somatosensory signals from muscle motion (flexion and extension) and convert them into distinguishable electrical signals. When the muscle contracts, a transfer of surface charges appears owing to different electrification performances, in which PTFE has a strong ability to gain electrons, while natural latex tends to lose them. By increasing the separation distance between the two tribolayers, induced positive charges will form on the LIG electrode, leading to an alternating current flow to an external circuit system. A detailed illustration of the charge transfer in one flex‐extend cycle is shown in Figure S4, Supporting Information.

Due to the stable physical properties and the high conductivity arising from its unique atomic arrangement, graphene is a promising candidate for high‐performance sensors.^[^ ^46^, ^47^ ^]^ The well‐defined LIG electrode was directly written on the PI sheet via a one‐step laser writing process, which was highly efficient and feasible for pattering. The LIG presented a highly loose and ordered‐porous morphology with a cross‐sectional thickness of ≈32 µm (Figure 2b and Figure S5g, Supporting Information). The resulting LIG structure was characterized by Raman spectroscopy, and the effect of laser power on LIG sheet resistance and thickness was also investigated (Figure 2c and Figure S5a,b, Supporting Information). After laser writing, flat PTFE tape was attached to the LIG electrode and served as the negative triboelectric material. PTFE is chosen due to its high toughness and strong electrification ability, showing better electrical output performance than other materials (Figure S5c, Supporting Information). Natural latex is selected as the positive electrification material because of its high air permeability, wear resistance, and skin‐friendliness. Specifically, one side of the natural latex provides a smooth surface for electrification, whereas the opposite side that contacts the skin exhibits a rough surface to reduce the possible motion artifact caused by slippage (Figure S5h,i, Supporting Information).

The electrical properties of the triboelectric sensor are crucial factors to be investigated. In order to quantitively characterize the output performances of the sensing unit, a linear motor was applied to exert periodic pressure as the external stimulus. Under an applied force of 1 N at varying frequencies from 0.5 to 2 Hz, the generated output signals are steady and uniform at each frequency, reflecting the high repeatability and reliability of the triboelectric sensor. The open‐circuit voltage (*V*_oc_) and short‐circuit transferred charge (*Q*_sc_) increase slightly, while the short‐circuit current (*I*_sc_) increases dramatically with a rise in frequency, from 1.54 to 10.84 nA (Figure 2d–f). We also evaluated the influence of the detached gap and contact area formed by the PET spacer. As shown in Figure S5d, Supporting Information, comparable voltage profiles under varied forces were recorded as the gap was retained at 0.1 to 0.5 mm. Consequently, the measured signals are almost the same in the low‐pressure region (<1 N), and exhibit higher sensitivities than in the high‐pressure region (>1 N). As the deformation of muscle/tendon only causes a slight displacement, a smaller gap is more suitable in practical applications.^[^ ^48^ ^]^ Furthermore, the voltage and current signals obtained with a force of 1 N at 1 Hz both increase with the larger area (variable diameter from 9 to 15 mm), from 0.30 to 1.43 V, and 1.58 to 5.87 nA, respectively (Figure S5e,f, Supporting Information). Although a larger sensor area can generate higher output, the size of the sensor is also restricted by the limited surface area of the wrist skin. Herein, the sensor size needs to be adjusted from person to person.

Mechanical durability and reproducibility are also important characteristics of on‐body sensors for long‐term use. Here a 10 000‐cycle durability test was performed to press the triboelectric sensor with a 1 N force at 2 Hz. As illustrated in Figure 2g, the current response of the triboelectric sensor is stable and repeatable, which presents a gradual increment (from 12.45 nA in the initial state to 15.84 nA at the end) after 5000 s of continuous pressing. The increased current with the working cycles may be attributed to the charge accumulation on the electrification surface of the triboelectric sensors.^[^ ^16^ ^]^ Upon loading the same pressure, both the response and recovery time appear to be very short, within 100 and 60 ms, respectively (Figure 2h). The fast response property further guarantees the effective capture of hand motion‐related triboelectric signals. Moreover, the long‐time stability of the triboelectric sensor was explored after storing the sensor in the dry environment for six months. Due to the functional materials with stable physical and chemical properties, the output performance of the triboelectric sensor shows negligible changes (Figure 2i).

To further assess the triboelectric sensor for retrieving muscle/tendon‐motion information, the signal quality acquired using our sensor was compared to the traditional commercial sEMG electrode (Ag/AgCl gel electrode). Figure 2j and Figure S6, Supporting Information, illustrate the placement of a triboelectric sensor and sEMG electrodes for detecting the movement of the anterior flexor muscles (green dots in Figure 1e). As observed, it only requires one triboelectric sensor placed on the palmar wrist (position 1), which is more convenient and skin‐friendly to use than the three sEMG electrodes firmly pasted around the forearm (position 1–3). The traditional sEMG methods could embody inefficiencies since a large number of sensors will increase the number of outer wirings, and also introduce time and labor‐intensive data preprocessing.^[^ ^4^ ^]^ Besides, too many wearing electrodes can cause skin discomfort and wearing burden. As shown in Figure 2k, the triboelectric signal presents a more distinctive feature than the sEMG signal. The average peak amplitude of triboelectric signals (≈320 mV) is 533 times higher than that of the sEMG electrode (≈0.6 mV) while performing the middle finger flexion gesture. Likewise, the signal quality of our sensor also shows a much higher signal‐to‐noise ratio (SNR) than that of the sEMG electrode, which is 43.1 and 22.2 dB, respectively (Figure 2l). In order to show the capability for monitoring steady‐state muscle motions, the subject was asked to grip three elastomer ringcollars with the strength of 20, 40, and 60 LB, respectively. As shown in Figure S6, Supporting Information, the steady‐state gripping is completely recorded, and higher signal amplitudes are consistent with the increased force levels. These results reveal that the high‐performance triboelectric sensors can reliably detect micro muscle/tendon contraction, enabling them to be suitable as desired receptors for the classification of dexterous hand motions.

### 2.3 On‐body Gesture Information Acquisition and Analysis

**Figure 3:**
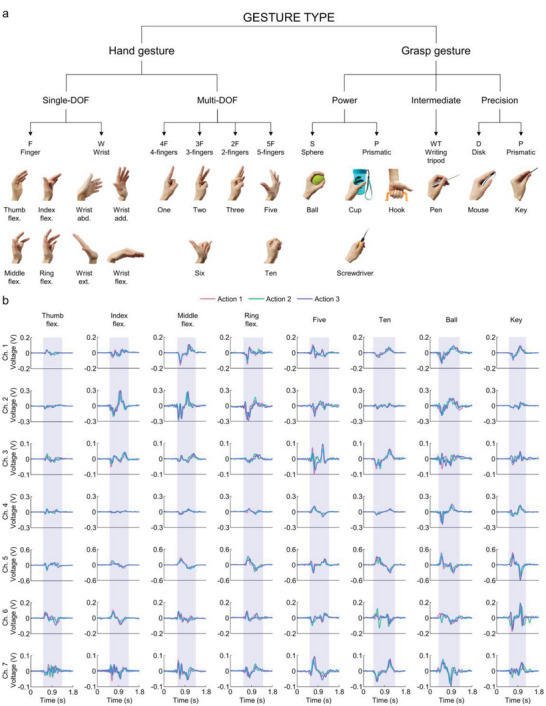
Hand motion dataset and triboelectric recording characteristics. a) Photographs showing the 21 categories of hand motions. The proposed taxonomy contains two top‐level categories: “hand gesture” and “grasp gesture.” The hand gesture subset is subdivided into single‐DOF (finger and wrist: F and W) and multi‐DOF gestures (multiple finger flexion and extension: 5F, 4F, 3F, and 2F). A further classification in the grasp gesture subset involves power (S and P), intermediate (WT), and precision grasps (D and P). b) Example raw waveforms recorded by the triboelectric smart wristband when performing eight different hand motions.

Using our gesture sensing system, we collected and explored a custom gesture dataset including 21 categories of hand motions. As shown in **Figure** **3**a, the dataset consists of the movements of multiple fingers and the wrist with different degrees of freedom (DOF). The “hand gesture” set contains the flexion (flex.) and extension (ext.) of different finger DOF, along with the rotation (flex. and ext.) and translation (abd., abduction; add., adduction) of the wrist joint. The “grasp gesture” set includes frequently used gestures in daily life for grasping objects, in which the gesture only uses distal and proximal interphalangeal (DIP and PIP) joints are defined as a precision grasp, while the gesture also involves palm and metacarpophalangeal joints are defined as a power grasp^[^ ^49^, ^50^ ^]^ (see Note S1 and Table S1, Supporting Information, for the details of grasp gestures).

To facilitate comparisons, we conduct preliminary data analysis to provide further insights into the proposed correlations between hand motions and their predominant muscle/tendon groups (the detailed relationship is demonstrated in Figure S7, Supporting Information, and the hand‐motion signal patterns are arranged in Figure S8, Supporting Information). It is noteworthy that most gestures involve several different kinds of muscle/tendon, and the collateral motion of nearby skin will also contribute to sensor signals. Figure 3b presents representative signal waveforms recorded from 7 channels while performing the flexion of the thumb, index, middle, and ring finger, the 5‐finger DOF gestures “five” and “ten,” as well as the “ball” and “key” grasp gestures. Each signal profile contains three independent actions, which last about 0.9 s (within shaded areas), and the action time slightly fluctuates (<0.2 s) from gesture to gesture. The signal patterns of the three actions are finely matched from channel to channel, indicating the stability and repeatability of the gesture sensing system. In contrast, there are noticeable differences in signals when performing gestures produced by different muscle/tendon activities.

The predominant muscle/tendon groups and sensor signals are closely correlated. Specifically, single‐finger‐DOF motions dominated by an independent muscle/tendon group exhibit the most distinguishable features. Take the flexion of the thumb, index, middle, and ring fingers as examples. While the thumb flexion is dominated by the pollicis muscle group (white dots in Figure 1e), the flexion of the other three digits is all dominated by the densely bounded anterior flexor muscle group (green dots in Figure 1e). As a result, the signal spectrums generated by the flexion of the index, middle, and ring fingers are relatively similar, presenting visible differences compared with that of the thumb. Besides, the maximum signal amplitudes of channels 1–3 (overlaying the anterior flexor muscles) are much lower when flexing the thumb, as the movements of pollicis muscle groups are far away from the corresponding sensors.

Hand motions activated by the same muscle groups can also be differentiated based on the temporal trend and intensity of the signal. Regarding the gestures of “five” and “ten,” both involve the anterior and posterior muscle groups, displaying similar maximum signal amplitudes but reverse signal waveforms. Since more fingers are involved in these two gestures, a more considerable degree of muscle contraction will be generated. Therefore, the signal intensity in some channels (such as channels 3 and 7, corresponding to anterior and posterior muscle groups, respectively) is more significant than that of the previous single‐finger‐DOF flexion. Additionally, the classification of grasp gestures depends on minute differences generated by grabbing forces and finger bending degrees. For instance, the “ball” gesture requires a forceful grip with strong muscle contraction, thus endowing the sensor array of intense stimuli to generate prominent signal patterns among all seven channels. Meanwhile, although it only takes a light force to perform the “key” gesture, the small and thin key reserves broad displacement space for the bending of phalanges, thus generating similar signal waveforms to the “five” gesture but with lower signal amplitude. Overall, these raw signal spectrums indicate the corresponding relationship between gestures and relevant muscle/tendon motions, and the close correspondence in the sensor signal is certainly beneficial for discriminating gestures. To further boost the efficiency and reliability of sophisticated gesture recognition, there is also a compelling desire for an advanced data analysis method to reveal the underlying information from a large number of databases.

### 2.4 Adaptive Accelerated Learning Algorithm and Classification Performances

**Figure 4:**
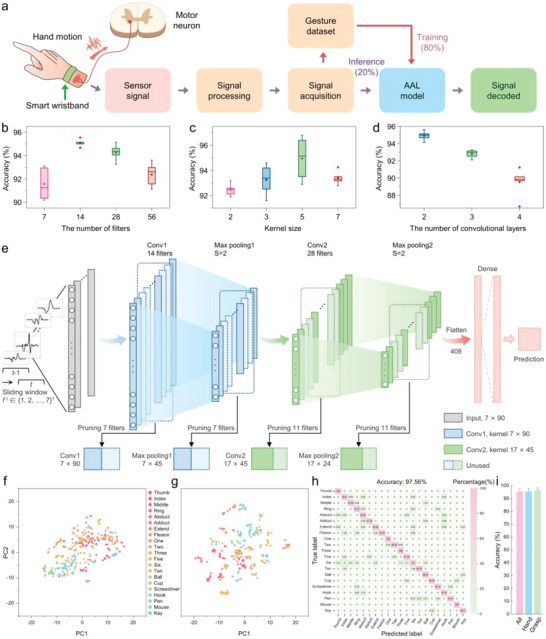
The adaptive accelerated learning model for gesture recognition. a) Deep‐learning‐aided data process flow, including training and inference processes mimicking the human brain. b–d) Comparisons of classification performances for the AAL structure parameters, including the number of filters, kernel size, and convolutional layers. e) Schematic diagram of the AAL model used for gesture recognition, in which the dotted boxes are discrete filters being pruned. f,g) Visualizing the gesture signals from the input and output layers using t‐SNE dimensionality reduction. h) Confusion matrix for hand‐motion signals generated from 21 gestures. i) Results of the multi‐subject experiment. Bars represent the average accuracy in different test groups.

**Figure** **4**a presents the flow diagram for training and inference of hand motions, analogous to the way that the brain interprets encoded body information via vast circuits of sensory receptors, neurons, and synapses. Unidentified signals detected by the triboelectric sensor array, which are relevant to defined hand motions, first go through signal processing before being collected into the gesture dataset (details are demonstrated in Figure S9, Supporting Information). Based on the received spectrum of training signals (80%), an AAL model classifier will then decode the new input signal (20%) for gesture recognition.

To implement the recognition task based on the inconstant triboelectric signals, we constructed the AAL architecture based on a 1D CNN model. For the backbone of CNN, the number of filters, kernel size, and convolutional layers were adjusted to realize optimum recognition performances. As illustrated in Figure 4b–d, the CNN model with 14 filters, kernel size of 5, and 2 convolutional layers possessed the optimal overall accuracy. Figure 4e depicts the schematic diagram of a two‐layer sub‐network constructed on the optimized CNN model. The 7‐channel gesture signals are projected into a space‐time map for both the training and inference processes, in which the time domain data are segmented by 900 ms sliding windows with 90% overlap. For the feature extraction in the training process, we introduce an adaptive pruning strategy for realizing self‐network sampling. The segmented data passed through convolutional layers will be filtered by channel‐wise discrete gates, which can dynamically prune the corresponding channels (and their associated filters, dotted boxes in Figure 4e) of less contribution on the output feature map. After pruning discrete filters, each convolutional layer and the corresponding max‐pooling layer is greatly compressed, with the number of filters being reduced from 14 to 7 and from 28 to 17, respectively. In particular, the importance of channels is variable with the small batch of training orders. Based on this, the state of discrete gates is kept updated in the training process for carefully selecting the most influential filters to adapt to different inputs. Therefore, with the probability of pruned filters being reactivated, the inference procedure is always under the most efficient condition based on the exceptionally compact and optimal pre‐trained model. Compared with previous works that needed to fine‐tune the unchangeable pruned models,^[^ ^51^, ^52^ ^]^ our adaptive filter pruning can effectively reduce the model size without additional treatment and maintain better performance. The detailed parameters in the data training process and AAL model are described in Note S2 and Table S2, Supporting Information.

To visualize the general clustering of the overall gesture dataset, we apply t‐distributed stochastic neighbor embedding (t‐SNE) in the 2D feature space. Different colors on the profile represent different gesture categories, and each point refers to the trial of one hand motion projected from the high‐dimensional dataset into two dimensions (principal component 1 and principal component 2). The two t‐SNE distributions of 21 classes for the input layer and the output layer are demonstrated in Figure 4f,g. These results indicate a desirable feature clustering after undergoing the AAL model, showing less overlap and stronger interclass separability. The feature clustering results prove that the proposed AAL model can properly classify the feature information. Figure 4h shows the confusion matrix for these 21 gestures with a high recognition accuracy of 97.56% over 50 trials from each motion (Figure S10, Supporting Information), while 90% of gestures have > 95% accuracy. It is noticed that the hand‐motion signals generated from similar gestures tend to have a higher confusion probability. For example, with 0.82% probability, “two” is predicted as “one” or “three.” In order to ensure the generalization ability of our gesture recognition system, seven volunteers are recruited, and two triboelectric smart wristbands are used in gesture data collection. Figure 4i demonstrates the overall recognition accuracy of different datasets obtained from seven subjects, showing a similar value with minor degradation as the number of gestures increases (from 96.67% over 7 grasp gestures to 95.52% over 14 hand gestures, and 95.41% over all 21 gestures). The detailed information and test accuracy curves in multi‐subject experiments are demonstrated in Section 4 and Figure S11, Supporting Information.

**Figure 5:**
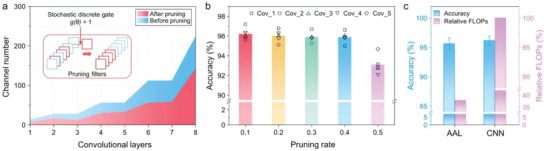
Characterizations of the pruning strategy. a) Channel configuration before (blue) and after pruning (red). Inset: Schematic showing the stochastic discrete gate‐dependent pruning model, in which the blue dotted boxes indicate the unimportant filters being pruned (with closed channels, *g*(*θ*) = 0), while the red boxes present the useful filters for training (with open channels, *g*(*θ*) = 1). b) Classification performances obtained by different pruning rates under five model configurations (cov_1 to cov_5; mean ± SD, *n* = 5, *p < 0.05, one‐way ANOVA). c) Comparisons of the performances of the AAL model and the original CNN model.

The self‐network sampling method is based on global pruning with probability for determining the pruned filters (Note S3, Supporting Information). Briefly, we use a stochastic discrete gate to represent the opening or closing of a channel, which is controlled by a learnable gate parameter *θ* that can be iteratively optimized according to the classification performances.^[^ ^53^ ^]^ Therefore, a channel always has the chance to be sampled by the sub‐network. As a result, a set of channels are carefully selected from each convolutional layer to reduce the model complexity, where the number of channels can be aggressively pruned to 50% of the original model (**Figure** **5**a). To obtain the pruned sub‐model with minimal model parameters and high recognition accuracy, only one global (non‐layer‐wise) hyper‐parameter is applied to control the pruning rate. As illustrated in Figure 5b, we conduct five model configurations to assess the effect of the pruning rate on their recognition accuracies in the training process (Note S2, Supporting Information). Desirable accuracies (>95%) are achieved by pruning rates ranging from 0.1 to 0.4, showing no statistical significance differences compared with the original model (*p*‐value > 0.05; Table S4, Supporting Information). Therefore, the maximum pruning rate is set to 0.4 to obtain the final AAL model. The comparisons between the performances of the resultant lightweight sub‐network and the original dense model are demonstrated in Figure 5c. Because of the reduced sampling features, the overall accuracy of the AAL sub‐network (95.87%) is slightly descended in contrast to the original CNN model (96.16%). To evaluate the complexity and computation costs of training models, we calculate the floating‐point operations (FLOPs) involved in the convolutional and dense layers. For the convolution layer, (FLOPs)_conv_ is defined as:

(1) $${\left(\mathrm{FLOPs}\right)}_{\mathrm{conv}}=\left(2\times C_{i}\times K^{2}-1\right)\times C_{o}\times H\times W$$

where *C*_i_ and *C*_o_ are the numbers of input and output channels, *K* is the kernel size, and *H* and *W* are the height and width of the output characteristic graph, respectively. For the dense layer, (FLOPs)_dense_ is defined as:

(2) $${\left(\mathrm{FLOP}\right)}_{\mathrm{dense}}=\left(2\times D_{i}-1\right)\times D_{o}$$

where *D*_i_ and *D*_o_ are the numbers of input and output neurons. The sum of (FLOPs)_conv_ and (FLOPs)_dense_ is taken as the final output. Consequently, the computation cost in the AAL sub‐network (0.51 × 10^6^) is significantly lower than that in the original CNN model (1.35 × 10^6^), indicating a large number of FLOPs (around 62.19%) has been successfully pruned to accelerate the prediction process. The comparison results present the high performances of the pruned architecture, which can significantly reduce the model's computation operands with minimal loss of identification accuracy.

### 2.5 Demonstrations of Multi‐Class Teleoperations

**Figure 6:**
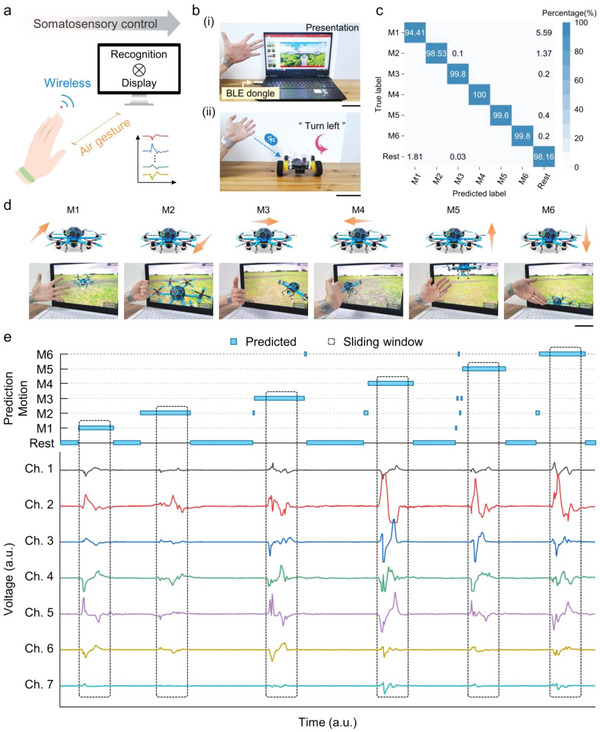
Demonstrations of wireless, real‐time HMIs. a) Schematic diagram showing the process of recognizing air gestures for somatosensory control. b) Scenes of the demonstrations of the control of i) presentation software and ii) bipedal vehicle. Scale bars, 10 cm. c) Confusion matrix for classifying seven hand motions. M1 to M6 refers to open hand, closed hand, wrist flexion, wrist extension, wrist adduction, and wrist abduction, respectively; Rest means no motion. d) Proof‐of‐concept demonstration of a VR game, showing the 3D drone control of moving forward, backward, right, left, up, and down, respectively. Scale bar, 10 cm. e) Predicted motions and corresponding triboelectric signals of M1 to M6.

In tandem with rapid progress in mobile networks and intelligent terminals, air gesture control brings a broad prospect for constructing more convenient and intuitive interactions than traditional handheld controllers. However, the current air gesture control is mainly based on visual information, which is susceptible to light conditions and blocking objects and will restrict the user's actions in the camera view. Hence, a general solution for projecting hand motions with high precision and low power consumption is needed to explore new avenues for next‐generation cyber‐human interfaces. As a proof‐of‐concept application, we demonstrated wireless, real‐time human–machine platforms based on the proposed smart wristbands. **Figure** **6**a shows the schematic process for somatosensory control via air gestures, allowing dynamic gesture identification and efficient parallel control of machine terminals. The computer acquires the hand motion‐related triboelectric signals via the communication protocol of the serial port (BLE dongle in Figure 6b‐i). Each of the four categories of air gestures (open hand, closed hand, and wrist flexion/extension) is assigned a specific command for freely manipulating a presentation software (Microsoft PowerPoint; Figure 6b‐i and Video S1). Moreover, the control commands can be transmitted to a bipedal vehicle via Bluetooth for accomplishing complex track motions (Figure 6b‐ii and Video S2). For instance, the signals motivated the differential steering of two wheels to turn left as the hand swung to the left. Details of the hand motions and intended commands for these two terminals are presented in Table S5, Supporting Information.

In addition, the emerging virtual reality/augmented reality (VR/AR) technologies have achieved drastic advances in various fields with the benefit of enhanced interactions. Smart wristbands have great potential for realizing an immersive user experience and improving the effectiveness of VR/AR experiences, owing to the great consistency between real and virtual activities. Here, six control classes are projected into a VR program, including the above four motions and wrist adduction/abduction. As shown in the confusion matrix in Figure 6c, after the training process in the AAL model, the recognition accuracy reaches 98.51% over 50 trials from each gesture. The 3D motions of a virtual drone in cyberspace are labeled according to target motions from M1 to M6 (Figure 6d and Video S3). The real‐time predicted results in each sliding window are consistent with time‐domain triboelectric signals (Figure 6e), exhibiting robust and reliable recognition performances. Furthermore, latency is also important in realizing immersive VR/AR experiences. Because of the sensing strategy of the sliding window algorithm and a proper threshold voltage, the prediction and hand motion were almost in synchrony rather than after the action was fully completed. The resultant response time is less than 1 s, including the time from detecting action to executing control commands to target machines. In addition, the appropriate window length can ensure sufficient signal information for classification, while avoiding misprediction and continuous instruction input. The context changes when the wristband was doffed and redonned (>1 h apart) in approximately the same location on the wrist was explored. As shown in Figure S13, Supporting Information, this resulted in an 18% accuracy degradation (from 98.59% to 80.59%). The decrease in accuracy may be caused by artificial factors, such as wearing tightness or wearing position deviation of the wristband. The classification for a combination hand motion of M4 and M5 was also investigated. As depicted in Figure S14, Supporting Information, the recognition accuracy reaches 99.47% over 50 trials from each gesture. This endows the possibility of decoupling combined gestures using our system. Overall, these results reveal the portable, self‐powered smart wristbands as promising multifunctional HMIs toward broader applications in intelligent robotics, work assistance, and the military field for executing dangerous missions remotely.

## 3 Conclusions

In this study, we have reported a self‐powered, wearable gesture sensing system based on triboelectric sensors and a lightweight deep learning model to realize high‐precision and highly‐efficient hand gesture recognition. The triboelectric sensor array can conformably attach to the human wrist with a wearable strap, transferring the muscle/tendon contractions into high‐quality electrical signals. By virtue of the surface charging effect, the sensor array can monitor muscle/tendon activities in a reliable, highly sensitive and cost‐effective approach that transcends the traditional sEMG method. Importantly, the close correspondence between the actions of dominant muscle/tendon groups and gestures leads to distinctive features in sensor signals, which can enhance the accuracy of gesture classification. Through the analysis of the collected information from different hand motions, the sensing system is proved to have great durability and repeatability, and a corresponding relationship is observed between signal waveforms and movements of relevant muscle/tendons.

An accelerated neural network is proposed for gesture recognition, which is optimized by an adaptive pruning model to boost the efficiency and reliability of the classification procedures. As a result, integrating the optimized machine learning algorithm and the intelligent wristband leads to successful classifications of 21 hand motions with a superior accuracy of 97.56% and a mean accuracy of 95.41% among 7 participants. Through leveraging the adaptive pruning strategy, the computing costs of the original model can be significantly reduced to one‐third while the test accuracy is hardly sacrificed. Combing the identification and motion‐detecting capabilities, the gesture recognition system is also implemented for wireless, real‐time control, and navigation of multiple terminals with a short time delay within 1 s.

In contrast to other state‐of‐art methods developed for wearable HGR (**Table** **1**), our sensing system is designed in a more portable and ergonomic form. Our system leverages minimum sensor configuration (only 7 sensors) to classify a wealth of sophisticated and similar gestures with higher test accuracy. Furthermore, compared with commercial products of similar functionalities (5DT Glove, US$1990 and weighs 595 g; CyberGlove, US$40000 and weights > 300 g; gForce armband, US$375 and weighs 78 g; Myo armband, US$149 and weighs 96 g), our system is more economically viable (less than US$30) for industrial production, and more lightweight (weighs about 32.5 g as described in Table S6, Supporting Information). With the low‐cost and low‐complexity assembly of the generic triboelectric wristband, the system presented is promising for tasks in a wider scope of applications involving intelligent robotics, remote sensing controls, rehabilitation training, and immersive XR scenarios.

**Table 1:** Comparisons of this work and other human gesture recognition systems

Ref.	Mechanism	Device	Method	Sensors	Gestures	Accuracy (%)	Data set	Sampling rate	Self‐powered
This work	Triboelectric	Wristband	AAL	7	21	97.56	7 healthy	100 Hz	√
[23]	Triboelectric	Glove	CNN	10	11	95.23			**√**
[24]	Triboelectric	Glove	SVM	5	11	98.63	4 deaf	500 Hz	**√**
[25]	Triboelectric	Glove	CNN	16	6	96			**√**
[26]	Triboelectric	Glove	CNN	15	50	91.3			**√**
[4]	Resistive	Surface	LSTM	1	8	96.2			×
[31]	Resistive/visual	Patch	BSV	5	10	100	10 healthy	20 Hz	×
[54]	Capacitive	Wristband	CNN	15	15	95.0	20 healthy		×
[55]	Barometric	Wristband	LDA	10	21	94	10 healthy		×
[38]	sEMG	Patch	KNN	3	7	98.6	5 healthy	20 Hz	×
[44]	sEMG	Surface	HDC	64	21	92.87	2 healthy	1 kHz	×
[30]	sEMG/IMU	Wristband	LDA	5	8	92.6	10 healthy		×
[33]	sEMG/IM	Armband	CNN	8	7	97.81	17 healthy	200 Hz	×

Note: IMU: inertial measurement unit; LSTM: long short‐term memory; BSV: bioinspired somatosensory‐visual; LDA: linear discriminant analysis; HDC: hyperdimensional computing.

## 4 Experimental Section

### Triboelectric Nanogenerator Sensor Fabrications

The PTFE tape (50 µm in thickness) and natural latex film (80 µm in thickness) were utilized as the triboelectric materials, PI films (100 µm in thickness) as the substrate and electrode material, PET film (100 µm in thickness) as the spacer, respectively. All these commercial materials were cleaned with deionized water, ethanol, and acetone and then dried with nitrogen before use. Each functional layer was cut into the same tadpole‐like shape (head, the diameter of 14 mm; tail, the diameter of 5 mm; connection, the rectangle of 2 × 1 mm). Laser cutting and scribing processes were conducted with a 10.6 µm 30 W CO_2_ laser cutter system (Guangzhou HZZ E‐Photo Technology Co. Ltd., ILS‐3 V) at a maximum scan rate of 1524 mm s^−1^.

First, the LIG electrode was directly written into a tadpole‐like pattern (head, the diameter of 11 mm; tail, the square of 2 × 2 mm; connection, the rectangle of 2 × 0.5 mm). The same scribing rate of 152.4 mm s^−1^ and 1000 pulses per inch (p.p.i.) density were adopted for all laser scribing processes. The laser power varied from 1.8 to 4.8 W with increments of 0.6 W was employed to realize the conversion of only near‐surface PI film to LIG. Later, the LIG pattern was cut off with the PI film, and then the LIG‐patterned PI was directly covered by the adhesive PTFE tape. In addition, the head of the PET film was designed into an annulus shape (outer diameter, 14 mm; inner diameter, 11 mm), followed by attaching the natural latex film via double‐sided tape. Notably, there was a small space (1 mm in length) at the top of the PET annulus to avoid forming an enclosed chamber. Finally, the two as‐prepared parts were assembled into the triboelectric sensor.

### Characterizations of the Sensing Unit

The surface and cross‐section morphology of LIG and natural latex film were characterized by SEM (Hitachi SU3900). Raman spectra of LIG were obtained by a Raman spectrometer (Renishaw, InVia‐Reflex) with 532‐nm laser excitation at room temperature and a laser power of 5 mW. The resistance of LIG electrode was measured via the four‐probe method (SX1934 SZ‐82). For the electric output measurements of the sensing unit, a computer‐controlled force motor (LinMot, PS01‐23 × 80‐R) and a commercial force gauge (Xiamen Enlai Automatic Technology Co. Ltd.) were used to control and record the external pressure, respectively. The open‐circuit voltage, short‐circuit current, and short‐circuit transferred charge of the triboelectric sensor were tested by a programmable electrometer (Keithley 6514). USB 6536 acquisition card (National Instruments) was adopted to collect the data. The real‐time data acquisition control and analysis were realized by customized LabVIEW programs.

### Wristband Configurations

The original wrist strap consisted of a silk ribbon and a movable buckle for adjusting the tightness. The fabricated triboelectric sensors for each wrist position were attached to the wristbands via 3 M 467 double‐sided adhesive tape. In order to locate the wearing position, the movable buckle was placed on the styloid process of the ulnar each time. The tension of the wristbands was adjusted accordingly to ensure that the wristbands could be firmly and comfortably worn on the wrist, and that there existed enough contact‐separation space between the skin and triboelectric sensors.

### Signal Recording and Processing

Hand motion‐related triboelectric signals were recorded by a double‐layer PCB. The upper layer of the PCB includes the operational amplifiers (TLV274CPWR) for signal conditioning and a power supply chip (TPS6513). In particular, the triboelectric signal of each sensing unit was recorded by an individual operational amplifier to avoid signal crosstalk. The bottom layer of the PCB includes a microcontroller unit CC2640R2F with an on‐chip Bluetooth system for wireless streaming raw triboelectric signals. The obtained raw triboelectric signals were filtered with Butterworth low pass filter (48 Hz) for further analysis. For the SNR comparison, the sEMG and triboelectric voltage signals were collected and analyzed by NI 6218 (National Instruments) and customized LabVIEW programs. The sEMG signals were filtered with Butterworth low pass filter (30–200 Hz) and fast Fourier transform notch filter (50 Hz). The SNR was calculated with home‐customized MATLAB code by comparing the power spectrum and background noise level.

### Gesture Recognition Experiments

Quantitative validation of the triboelectric smart wristbands was conducted on 7 subjects (1 female and 6 males; Table S3, Supporting Information): body mass index of 16.4–23.3, wrist girth of 13.7–16.2 cm. According to the wrist girth of each subject, two kinds of sensor sizes were used to collect the triboelectric signals; small size (S) for the wrist girth of 14 ± 0.5 cm, and large size (L) for the wrist girth of 16 ± 0.5 cm. Subjects were asked to perform a total of 21 different hand motions in Figure 3a. In the rest position, participants comfortably kept their forearm on the table and naturally relaxed the fingers and wrist, avoiding elbow deviation and rotation. During each set, participants were told to begin the motion within a 3 s transition window, which involved the transient and non‐stationary signal. After the 3 s preparation, the participant performed the corresponding gesture and immediately (within 1 s) returned to the rest position to obtain the action signal. In order to avoid muscle fatigue, there is a 3 s of time interval between each action. Participants performed each gesture 5 times during each set and repeated 10 sets with a resting interval (1 min) in between. The study was conducted following the approved IRB protocol (IORG, No: IORG0003571) at Tongji Medical College, Huazhong University of Science and Technology. All participants agreed with the study procedures and provided signed consent forms.

### Statistical Analysis

Experimental results for classification accuracies obtained by different pruning rates are expressed as mean ± standard deviation (SD). There were 5 samples in the experimental results used in statistical analyses and they were analyzed by one‐way ANOVA, where *p < 0.05 was considered statistically significant. For all tests, the results were obtained by Microsoft Excel.

## Conflict of Interest

H.F. and H.W. are the authors of a patent application related to this work, filed with the State Intellectual Property Office of the P. R. China (application no. 202110439216.8; filed on 23 April 2021).

## Supporting information

Supporting Information

Supplemental Video 1

Supplemental Video 2

Supplemental Video 3

## Data Availability

The data that support the findings of this study are available from the corresponding author upon reasonable request.
