# Supplementary material for: Anatomically Designed Triboelectric Wristbands with Adaptive Accelerated Learning for Human–Machine Interfaces
Source: Adv Sci (Weinh). 2023 Jan 22;10(6):2205960. doi: 10.1002/advs.202205960 (PMC9951357; doi:10.1002/advs.202205960)
Supplement: Supplementary file 1 — Supporting Information [file ADVS-10-2205960-s002.pdf]

Supporting Information

Anatomically designed triboelectric wristbands with adaptive accelerated learning for human-machine interfaces

Han Fang, Lei Wang, Zhongzheng Fu, Liang Xu, Wei Guo, Jian Huang, Zhong Lin Wang,* and Hao Wu**

H. Fang, W. Guo, Prof. H. Wu
Flexible Electronics Research Center, State Key Laboratory of Digital Manufacturing Equipment and Technology, School of Mechanical Science and Engineering, Huazhong University of Science and Technology, Wuhan 430074, China
E-mail: hwu16@hust.edu.cn

L. Wang, Z. Fu, Prof. J. Huang
Ministry of Education Key Laboratory of Image Processing and Intelligent Control, School of Artificial Intelligence and Automation, Huazhong University of Science and Technology, Wuhan 430074, China
E-mail: huang_jan@mail.hust.edu.cn

Prof. L. Xu, Prof. Z.L. Wang
Beijing Institute of Nanoenergy and Nanosystems, Chinese Academy of Sciences, Beijing 101400, China
Prof. Z.L. Wang
School of Materials Science and Engineering, Georgia Institute of Technology, Atlanta, GA 30332-0245, USA
E-mail: zhong.wang@mse.gatech.edu

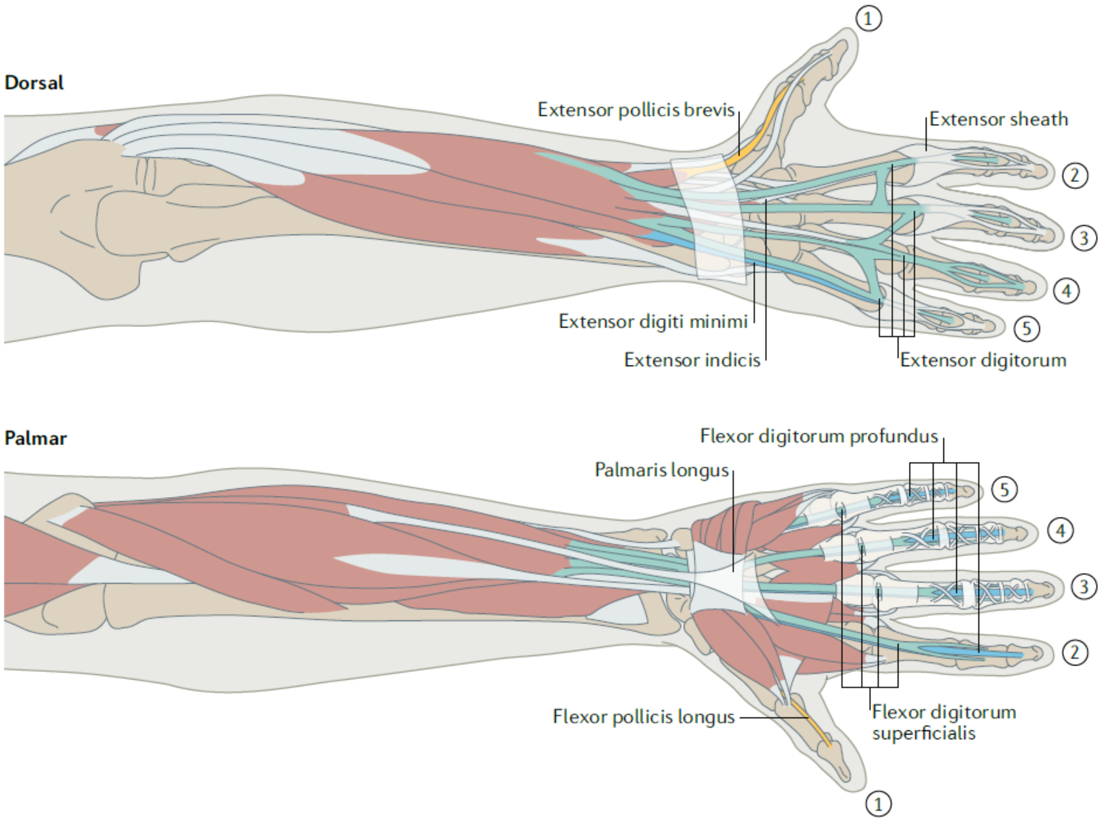

Figure S1. Human hand musculature. The hand is articulated by numerous muscle networks that cover the entire forearm, exhibiting complex paths, especially around the wrist, wrapping around multiple moving muscles and bones. Therefore, hand gestures are difficult to be estimated. Reproduced with permission from REF.[1], Springer Nature.

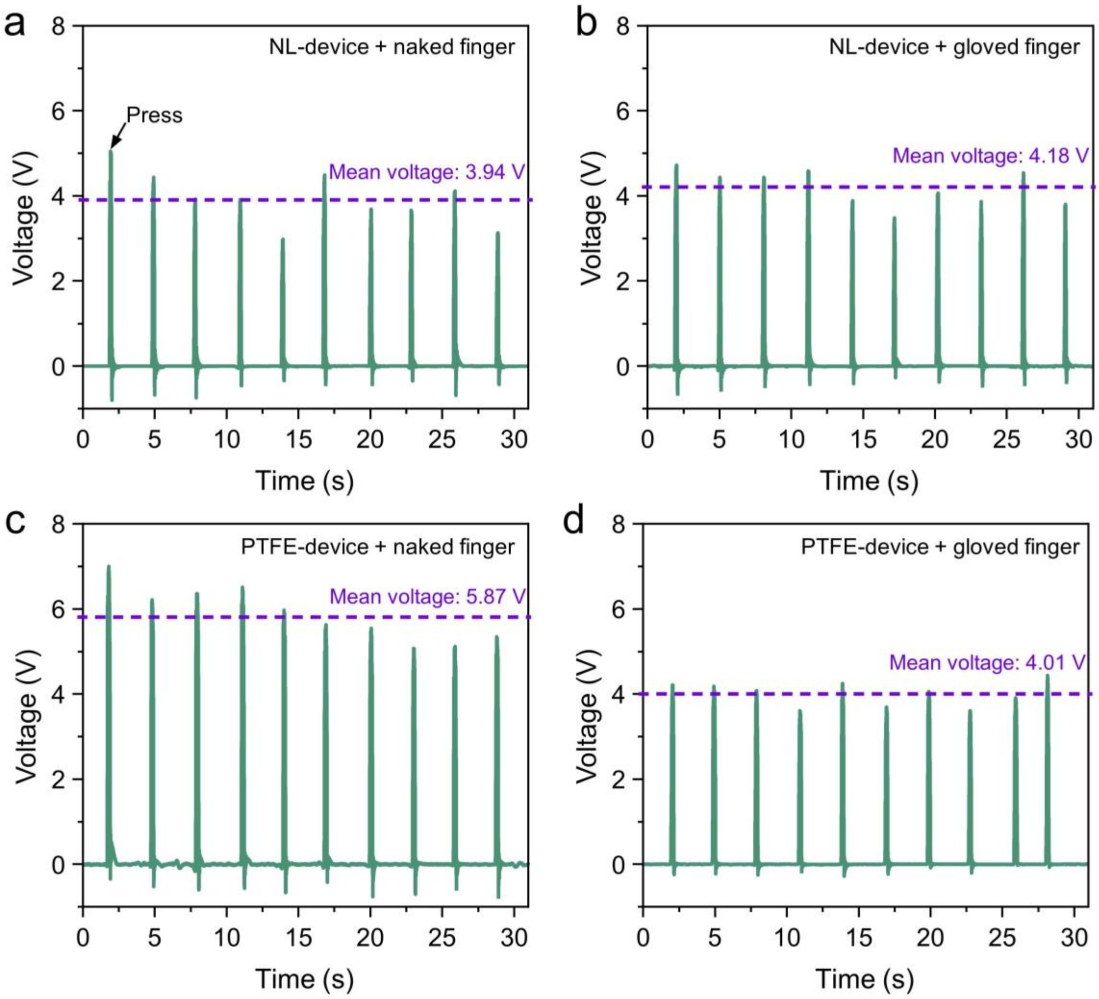

Figure S2. Effect of the skin on output performances. The open-circuit voltage of the device with the protection of natural latex (NL-device) pressed by a) a naked finger or b) a gloved finger. The open-circuit voltage of the device without the protection of natural latex (PTFE-device) pressed by c) a naked finger or d) a gloved finger. The electrical signal was acquired by the USB 6218 acquisition card (National Instruments).

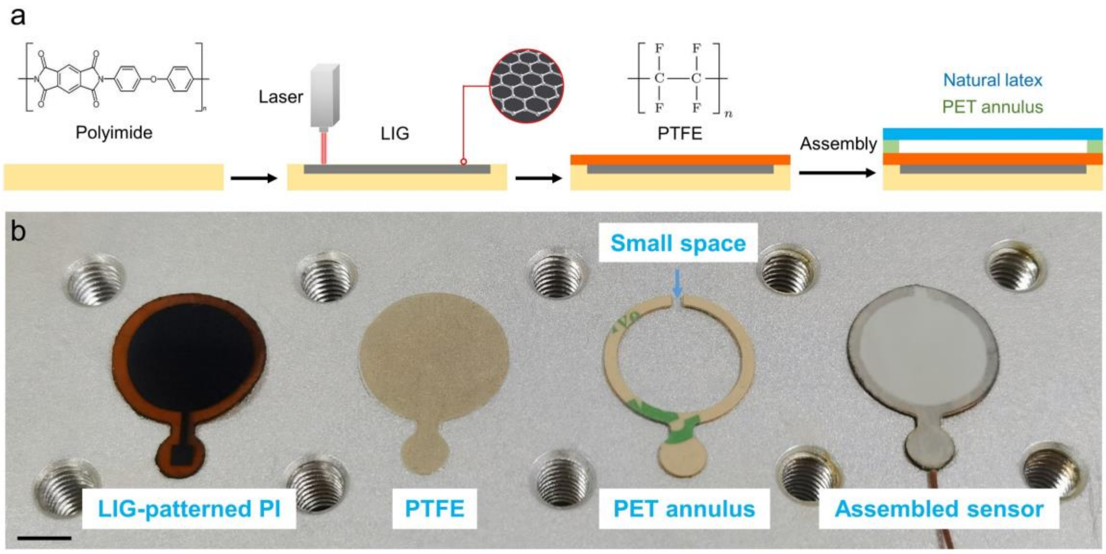

Figure S3. Fabrication processes of the triboelectric sensor. a) Schematic diagram showing the fabrication procedures for the triboelectric sensor. b) Photographs of the main components and the assembled sensor. Patterned adhesive tape was attached to the transparent PET annulus for better visibility. Scale bar, 5 mm.

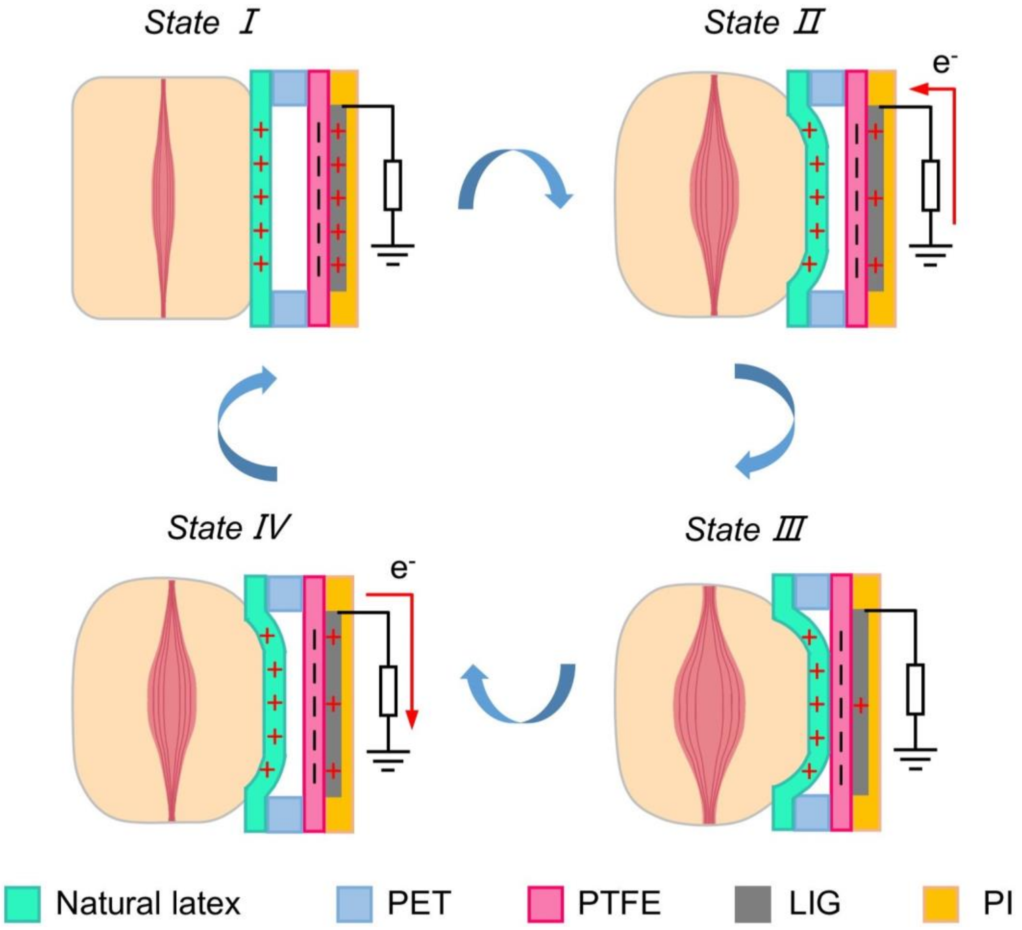

Figure S4. Schematic illustrations of the charging behavior of the TENG sensor when the muscle is in different states. *State I*: Initial stage with no charge changes, and the PTFE and natural latex films induce positive charges on the LIG electrode. *State II*: In the muscle contraction stage, the natural latex film is pushed closer to the PTFE film. As the induced charges on LIG electrode gradually decrease, the electrons flow to the LIG electrode from the ground in the external circuit system. *State III*: When the muscle contraction reaches its maximum, the induced charges on the electrode reduce to almost zero. *State IV*: When the muscle gradually releases, there is an opposite electron flow in the external circuit system until the muscle reaches its initial condition *State I*.

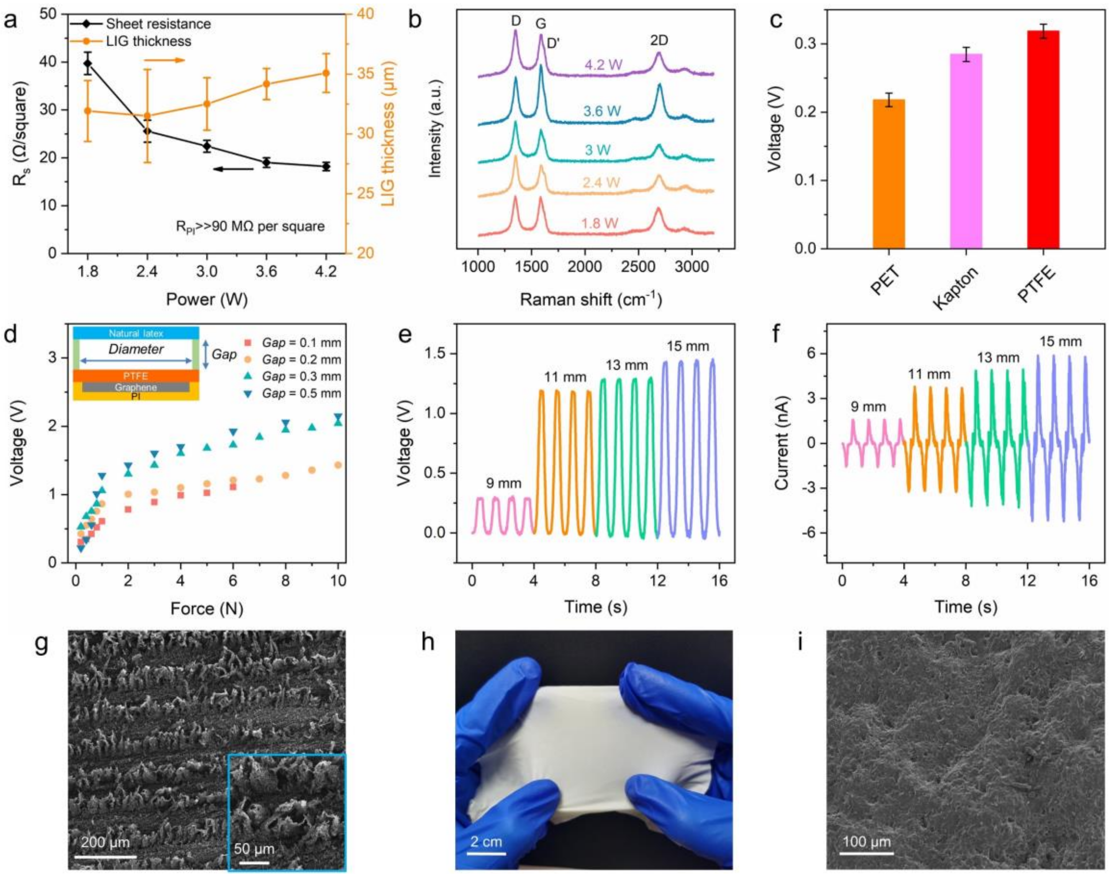

Figure S5. Characterizations of the TENG sensor. a) Correlations of the sheet resistance (R_S) and LIG thicknesses with the laser power. Generally, a higher laser power will result in lower R_S and a thicker LIG sheet. b) Raman spectra of LIG films obtained with different laser power. c) The shift voltage of three different materials as friction pair with natural latex. d) The output open-circuit voltage of the TENG sensor with different gap distances. e and f) Open-circuit voltage and short-circuit current output obtained from TENG sensors with different gap distances at a frequency of 1 Hz with a force of 1 N. g) SEM image of the LIG film. Inset: the corresponding higher magnification SEM image. h) Photo of the rough side of the natural latex film. i) The corresponding SEM image of the rough side of the natural latex film.

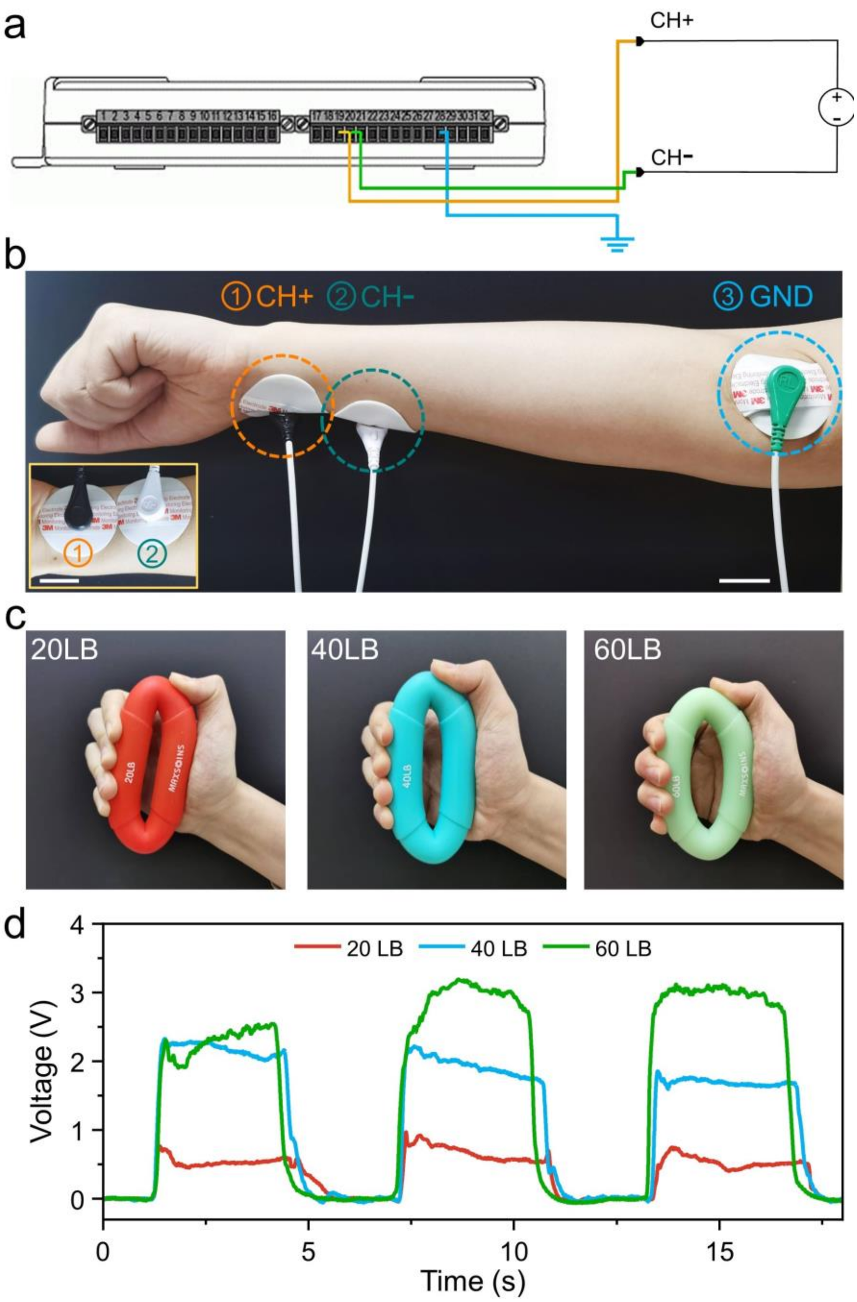

Figure S6. Measurement method of sEMG signal. a) Schematic diagram showing the wire connections of sEMG electrodes in a LabVIEW program. b) Photos showing the wearing positions of three sEMG electrodes. Inset: Photo of the two differential electrodes. Scale bars, 2 cm. The potential difference is determined by two exposed electrodes (electrodes 1 and 2) pasted on the palmar side. Electrode 3 pasted on the elbow (dorsal side) is for ground connection. c) Photographs and d) plots showing the steady-state signatures of hand grips at force levels of 20 LB, 40 LB, and 60 LB, respectively.

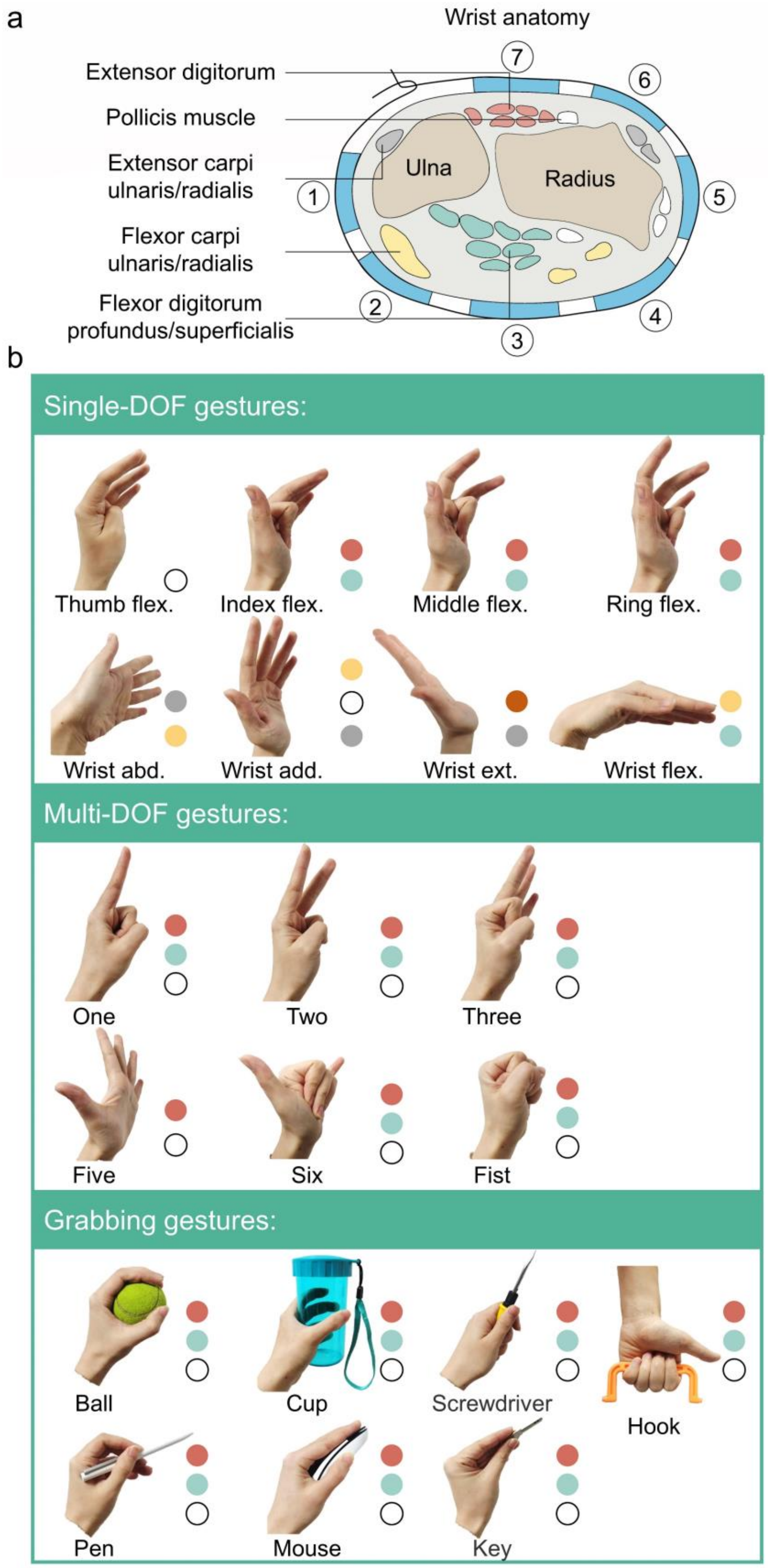

Figure S7. The corresponding relationship between gestures and muscle/tendon groups. a) Cross-section anatomy of the human wrist, and the colored dots in b) refer to the muscle/tendon groups shown in a).

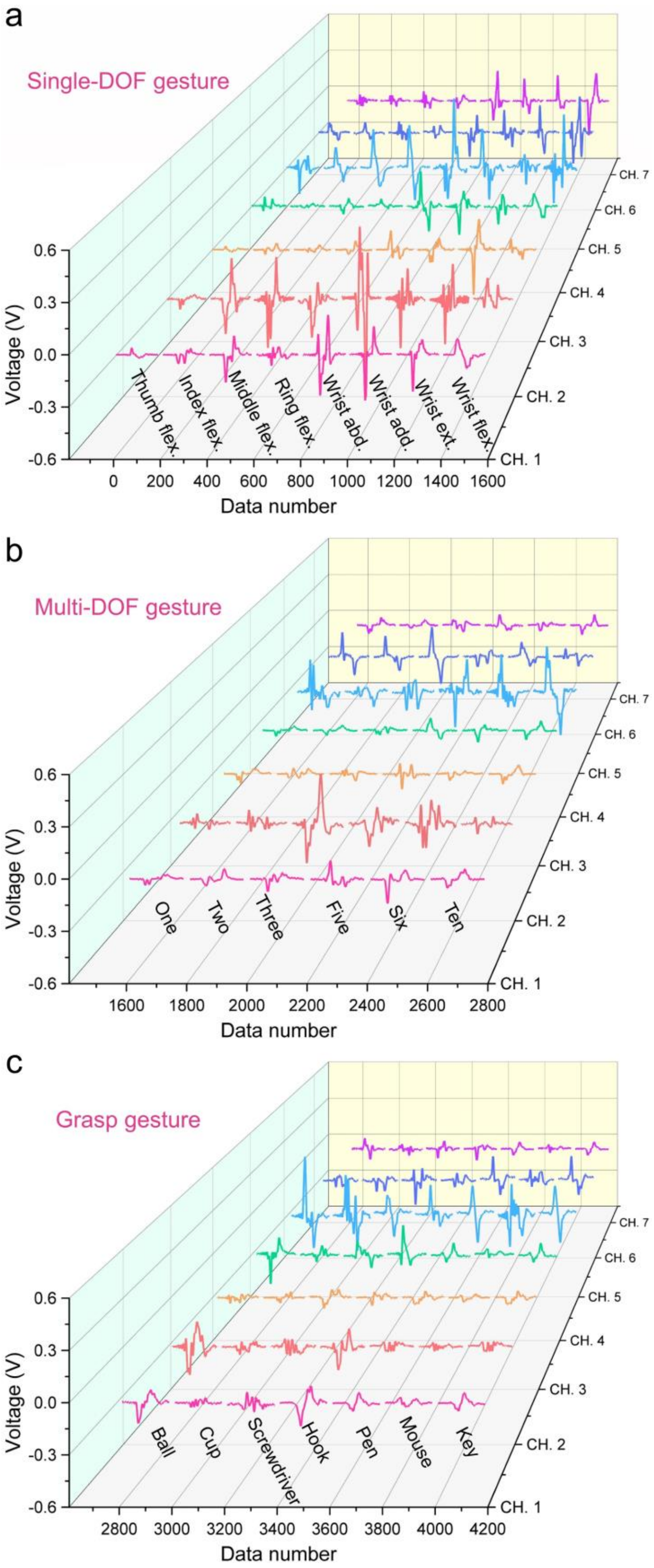

Figure S8. The hand-motion voltage waveforms of 21 gestures. a) Single-DOF hand gestures, b) multi-DOF hand gestures, and c) grasp gestures.

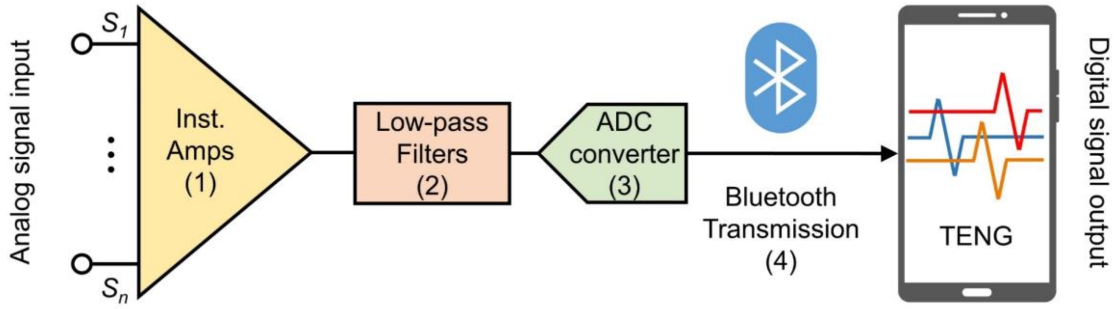

Figure S9. Flow chart showing the triboelectric signal processing and acquisition processes. The signal process circuit comprising of four main parts: (1) instrumentation amplifiers to amplify the obtained signals, (2) 30-Hz low-pass filters to avoid power-line interference, and (3) an analog-to-digital converter (ADC) to convert the analog signals into digital signals before being wireless sent to a terminal display via (4) the Bluetooth.

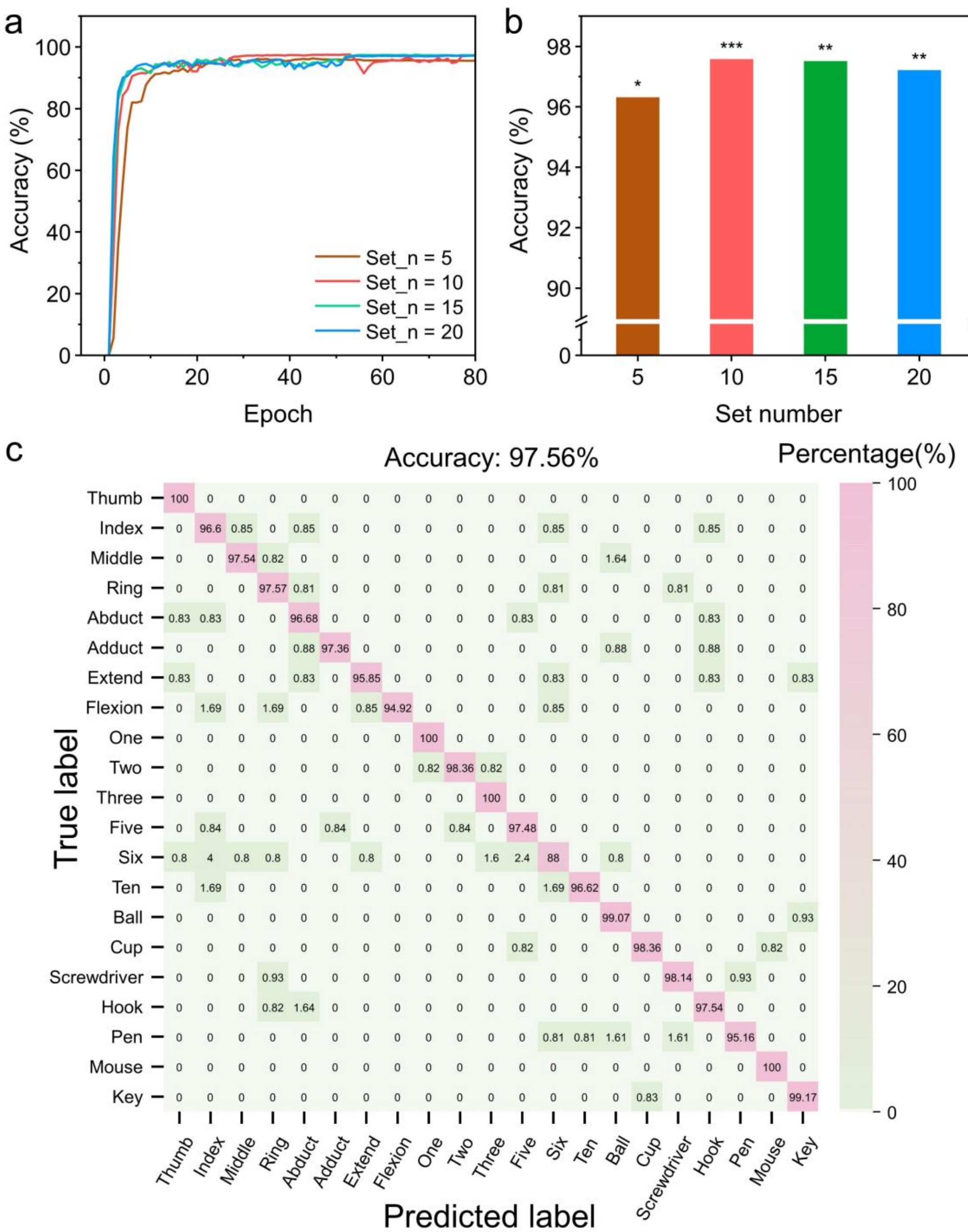

Figure S10. The test accuracy of 21 hand motions with different training set numbers. a) The accuracy curves of the AAL model with different numbers of training sets. b) The test accuracy with different numbers of training sets. After more than 10 training sets, the accuracy reaches an acceptable level (> 97%). c) Confusion matrix with 10 training sets.

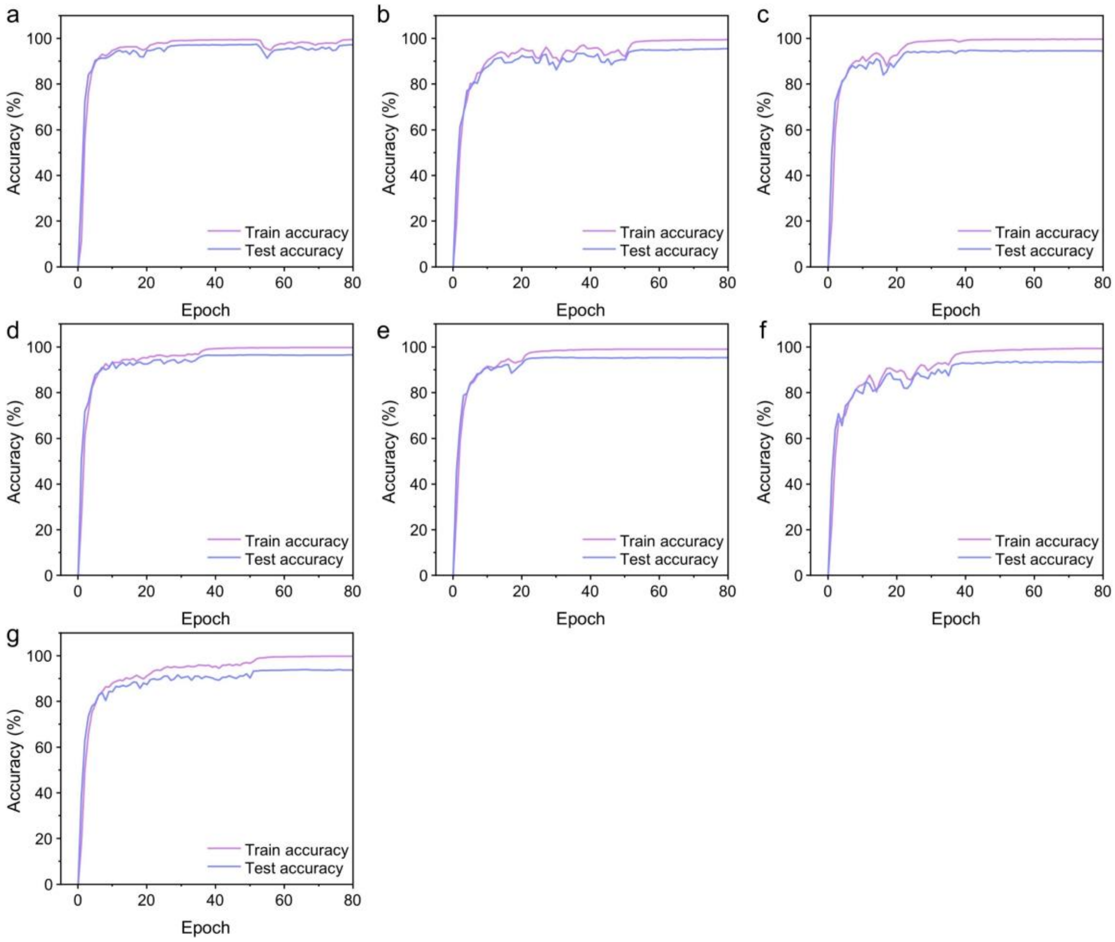

Figure S11. The training and test accuracy increase with epochs. a-g) The accuracy curves of the AAL model when training 21 hand motions performed by subjects #1 to #7.

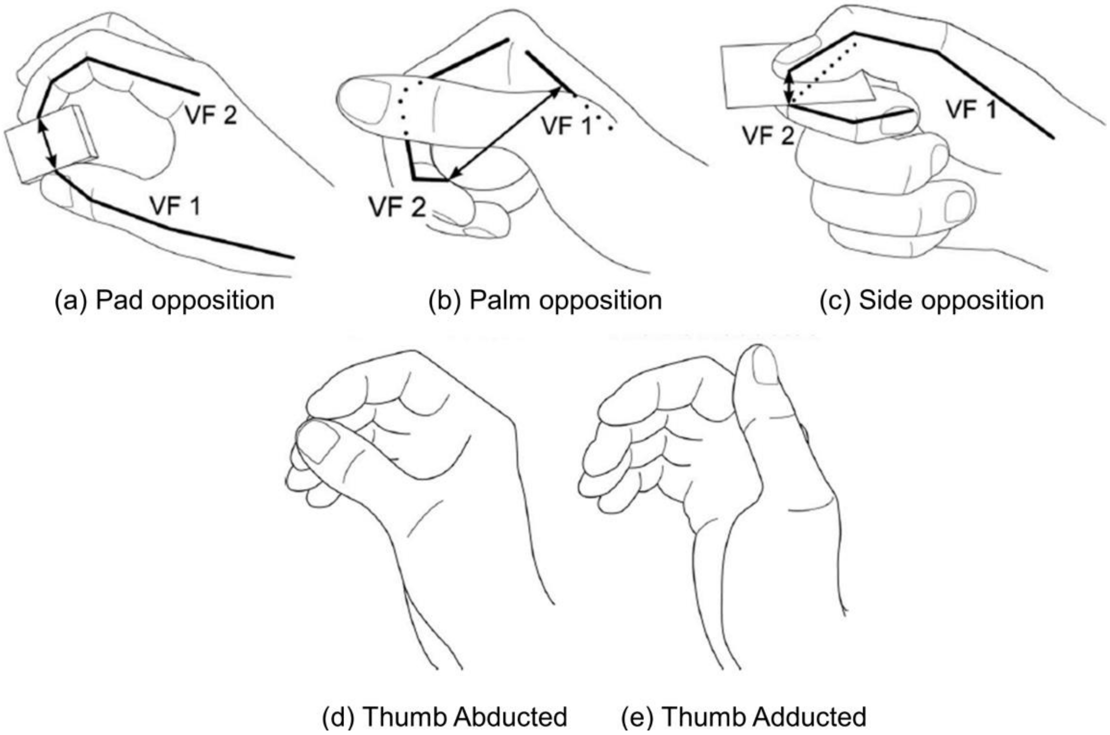

Figure S12. Opposition types of the grasping hand. According to the force direction applied by a functional unit involving several fingers (virtual finger (VF)) or hand parts, there are three basic directions: a) pad, b) palm, and c) side oppositions, where the hand surface are generally parallel, perpendicular, or transverse to the palm, respectively. Moreover, the thumb can either e) be abducted or e) adducted to oppose the fingertips or “move away”. Reproduced with permission from REF.[2], © 2016 IEEE.

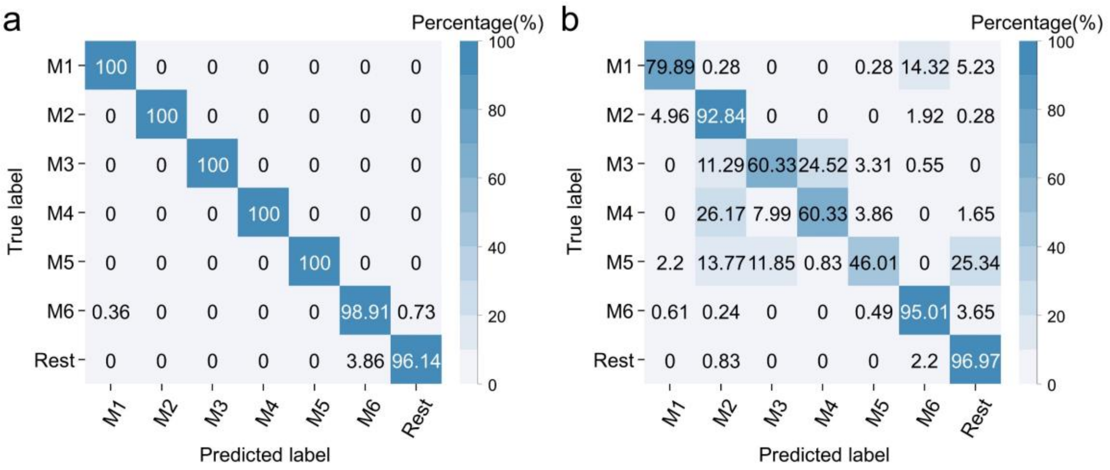

Figure R13. Confusion matrix for the classification of seven hand motions a) before and b) after doffing and re-donning (> 1 h apart) the wristband.

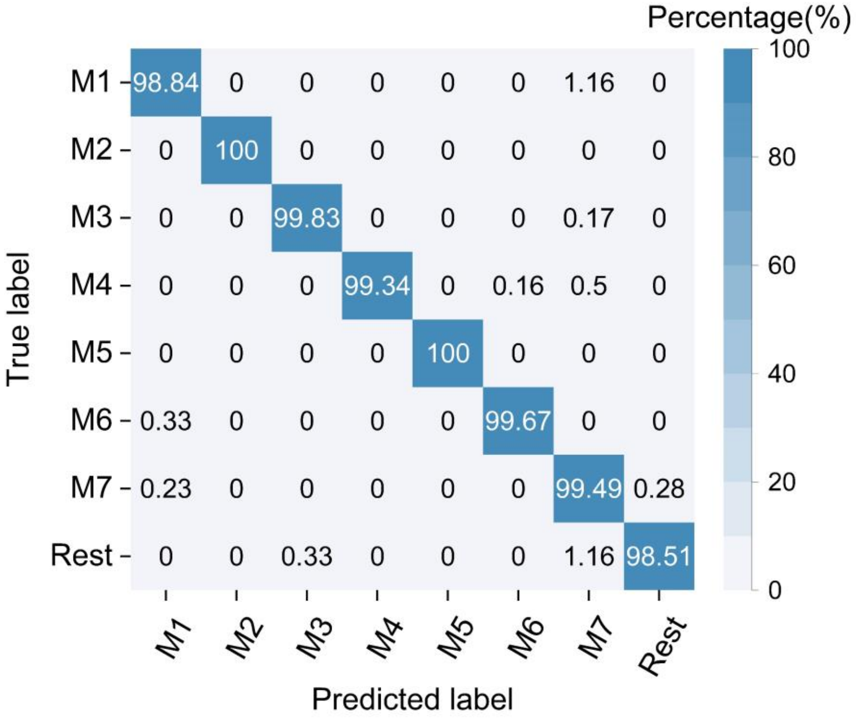

Figure R14. Confusion matrix for the classification of eight hand motions. M7 refers to the combination gesture of M4 and M5, i.e., the hand is extended and adducted 45°.

Note S1. The grasp taxonomy of different grasping gestures

The grasp classification in this work depends not only on the hand pose, but also on the shape of the object and the type of opposition between hand and object. For instance, "ball" (#1) and "cup" (#2) gestures have a similar handshape and object size, but the object in the former is a prism, while that in the latter is a uniform sphere. Therefore, the finger bending degree will be different. Besides, the thumb carpometacarpal (CMC) joint is adducted only in "screwdriver" (#3) and "hook" (#4) gestures, where the thumb exerts forces on the finger side (#3) or does not contact the object (#4). In contrast, thumb abduction is to act against other fingertips. Different from the four power gestures with the palm involved (called palm opposition), which has to be motivated by the arm, the grasping gestures where the thumb opposes the index finger are divided into pad opposition (#6 and #7) and side opposition (#5). For precision or intermediate gestures, the hand can implement intrinsic movements on the object without using the arm.

Overall, the definition of different oppositions is demonstrated in Supplementary Figure 11. The properties of each grasping gesture, including the major size of objects, force type, and opposition type, are listed in Supplementary Table 1.

Note S2. Setup of the data training process

For the data training process, we use stochastic gradient descent (SGD) with a data batch size from 300 to 800 with an internal of 100 to generate different model configurations, and an epoch number of 80 for iteratively updating model parameters, such as the gate parameter θ for determining filter pruning. In order to accelerate the training procedure, the learning rate is reselected every 10 epochs. To prevent the training model from overfitting, a weight decay of 0.0001 is used.

Note S3. Method of updating discrete gate parameters

The GPP strategy is used to ensure discrete gates with micro gate parameters can also

be reactivated as candidate channels again. Specifically, the GPP strategy for optimizing sub-network is guided by the macro-control of the loss function L_{pr}, which is defined as:

$$L_{pr} = \mu[\sum_{i=1}^{I}\sum_{j=1}^{J_i} g(\theta_j^i) - p_r \sum_{i=1}^{I}\sum_{j=1}^{J_i} 1^T]^2$$

where μ is a hyperparameter for balancing the cross-entropy loss and model compression loss L_{pr}, p_r is the pruning rate, $\mathbf{1} = [1,\ldots,1]^T$ is a vector with all ones, I represents the number of convolutional layers, and there are J_i channels in the i-th convolution layer. In the j-th channel from the i-th layer, a random function is used to generate a random number within [0, 1], and θ_j^i is clipped to be in the range of [0, 1] as the gate parameter. In each iteration, the comparison between the random number and θ_j^i is applied to define the stochastic discrete gate, which is enabled by using stochastic rounding:

$$g\left(\theta_j^i\right) = \begin{cases} 1 & w.p.\ \ \theta_j^i \\ 0 & w.p.\ \ 1-\theta_j^i \end{cases}$$

where *w.p.* means the with probability for reactivating the pruned filters. For updating θ_j^i, straight-through estimation (STE)[3] is used to directly transfer the gradient of $g\left(\theta_j^i\right)$ to θ_j^i as the original value, in which the θ_j^i that does not conform to the chain rule can also participate in the training process. θ_j^i is learnable according to the identification performances in the iterative process–that is, if the removal of a channel leads to a poor recognition accuracy, which means this channel could be important, θ_j^i will be increased and this channel is more possible to be sampled in the next iteration; In contrary, if the removal of a channel leads to a desirable recognition accuracy, θ_j^i will be decreased and this channel is more possible to be pruned in the next iteration.

The multiplication of $g\left(\theta_j^i\right)$ and the corresponding channel output is used as the

final output of the channel in the sub-network. As a result, a specific pruning rate can be obtained by minimizing L_{pr} . The opened discrete gates will not affect the corresponding parameters trained in the original network, while the parameters associated with the closed discrete gates will not participate in training.

Table S1. The properties of each grasping gesture.

#	Object	Grasp gesture	Mass [g], Diameter [cm]	Force type	Opposition type	Thumb position
1	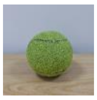 Ball	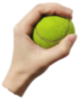	55, 6.3	Power	Palm	Abd.
2	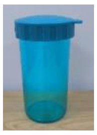 Cup	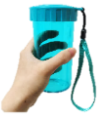	109, 6.5	Power	Palm	Abd.
3	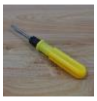 Screwdriver	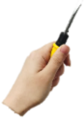	25, 2	Power	Palm	Add.
4	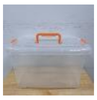 Box handle	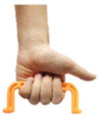	400, 1.5	Power	Palm	Add.
5	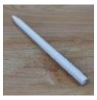 Pen	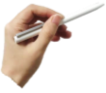	10, 0.9	Intermediate	Side	Abd.
6	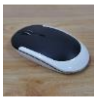 Mouse	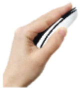	80, N/A	Precision	Pad	Abd.
7	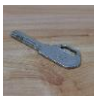 Key	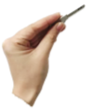	6, N/A	Precision	Pad	Abd.

Table S2. AAL model parameters. The detailed parameters for constructing the gesture recognition architecture.

No.	Layer type	No. of filters		Kernel/ Pool size	Stride	Input size	Output size	Padding
		Before pruning	After pruning					
1	Convolution 1	14	7	5	1	(None, 90, 7)	(None, 90, 7)	Same
2	Max pooling 1			2	2	(None, 90, 7)	(None, 45, 7)	Same
3	Convolution 2	28	17	5	1	(None, 45, 7)	(None, 45, 17)	Same
4	Max pooling 2			2	2	(None, 45, 17)	(None, 24, 17)	Same
5	Flatten					(None, 24, 17)	(None, 408)	Same
6	Dense					(None, 408)	(None, 21)	

Table S3. Biometric information and gesture recognition results of the participants.

Subject	Gender	Age	Wrist girth [cm]	Body mass index	Wristband size	All gestures (%)	Hand gestures (%)	Grasp gestures (%)
#1	Female	26	13.7	20.0	S	97.56	96.88	97.75
#2	Male	25	15.9	21.0	L	95.56	97.59	95.34
#3	Male	23	16.2	21.3	L	94.96	96.95	95.5
#4	Male	24	14.5	19.0	S	96.64	95.67	98.25
#5	Male	23	16.0	23.3	L	95.48	93.8	98.38
#6	Male	23	15.5	22.8	L	93.68	93.4	95.75
#7	Male	24	14.4	16.4	S	93.96	94.38	95.75
Average		24	15.2	20.5		95.41	95.52	96.67

Table S4. Accuracy and *p*-values of hyperparametric comparisons of different pruning rates.

**Pruning rate**	**Accuracy (%)**					**Overall accuracy (%)**	***p*-value**
	**Cov_1**	**Cov_2**	**Cov_3**	**Cov_4**	**Cov_5**		
0 (original model)	96.83	97.09	95.35	95.51	96.04	96.16 ± 0.60	
0.1	97.19	95.57	95.93	96.38	95.93	96.2 ± 0.39	0.9375
0.2	96.78	95.12	96.07	96.31	95.79	96.01 ± 0.38	0.7439
0.3	96.7	95.26	95.88	95.79	95.82	95.89 ± 0.27	0.5294
0.4	96.61	94.99	96.04	95.86	95.85	95.87 ± 0.34	0.5167
0.5	93.14	92.84	92.7	92.11	94.67	93.09 ± 0.92	0.0005

Table S5. List of control commands for the different terminals. A PowerPoint navigator, virtual drone, and RC car are controlled by the triboelectric signals from six gestures (five, ten, wrist flex., wrist ext., wrist add., and wrist abd.).

Gesture \ Terminal	PPT	Virtual drone	RC car
Five	Start PPT	Go forward	Forward
Ten	Exit PPT	Go backward	Backward
Wrist flexion	Next slide	Strafe right	Rotate right
Wrist extend	Previous slide	Strafe left	Rotate left
Wrist adduct	N/A	Move up	N/A
Wrist abduct	N/A	Move down	N/A

Table S6. The weight of each component of the hand gesture recognition system.

Sensor array	PCB	Battery	Wristband	Total weight
2.1 g	20 g	9 g	1.4 g	32.5 g

References

[1] A.R. Sobinov, S.J. Bensmaia, *Nat. Rev. Neurosci.* **2018**, *22*, 741.

[2] T. Feix, J. Romero, H. Schmiedmayer, A.M. Dollar, D. Kragic, *IEEE Trans. Hum. Mach. Syst.* **2016**, *46*, 66.

[3] M. Courbariaux, I. Hubara, D. Soudry, R. El-Yaniv, Y. Bengio, *arXiv preprint arXiv:1602.02830*, **2016**.
